# Autoinflammation in patients with leukocytic CBL loss of heterozygosity is caused by constitutive ERK-mediated monocyte activation

**DOI:** 10.1172/JCI181604

**Published:** 2024-10-15

**Authors:** Jonathan Bohlen, Ivan Bagarić, Taja Vatovec, Masato Ogishi, Syed F. Ahmed, Axel Cederholm, Lori Buetow, Steicy Sobrino, Corentin Le Floc’h, Carlos A. Arango-Franco, Luis Seabra, Marine Michelet, Federica Barzaghi, Davide Leardini, Francesco Saettini, Francesca Vendemini, Francesco Baccelli, Albert Catala, Eleonora Gambineri, Marinella Veltroni, Yurena Aguilar de la Red, Gillian I. Rice, Filippo Consonni, Laureline Berteloot, Laetitia Largeaud, Francesca Conti, Cécile Roullion, Cécile Masson, Boris Bessot, Yoann Seeleuthner, Tom Le Voyer, Darawan Rinchai, Jérémie Rosain, Anna-Lena Neehus, Lucia Erazo-Borrás, Hailun Li, Zarah Janda, En-Jui Cho, Edoardo Muratore, Camille Soudée, Candice Lainé, Eric Delabesse, Claire Goulvestre, Cindy S. Ma, Anne Puel, Stuart G. Tangye, Isabelle André, Christine Bole-Feysot, Laurent Abel, Miriam Erlacher, Shen-Ying Zhang, Vivien Béziat, Chantal Lagresle-Peyrou, Emmanuelle Six, Marlène Pasquet, Laia Alsina, Alessandro Aiuti, Peng Zhang, Yanick J. Crow, Nils Landegren, Riccardo Masetti, Danny T. Huang, Jean-Laurent Casanova, Jacinta Bustamante

**Affiliations:** 1 Laboratory of Human Genetics of Infectious Diseases, Necker Hospital for Sick Children, Paris, France.; 2 Paris Cité University, Imagine Institute, INSERM U1163, Paris, France.; 3 Heidelberg University, Heidelberg, Germany.; 4 St. Giles Laboratory of Human Genetics of Infectious Diseases, The Rockefeller University, New York, New York, USA.; 5 Cancer Research UK Scotland Institute, Glasgow, United Kingdom.; 6 Science for Life Laboratory, Department of Medical Biochemistry and Microbiology, Uppsala University, Uppsala, Sweden.; 7 Laboratory of Chromatin and Gene Regulation during Development, Paris Cité University, INSERM U1163, Imagine Institute, Paris, France.; 8 Laboratory of Human Lymphohematopoiesis, INSERM U1163, Imagine Institute, Paris, France.; 9 Primary Immunodeficiencies Group, Department of Microbiology and Parasitology, School of Medicine, University of Antioquia, Medellín, Colombia.; 10 Unit of Allergy and Pneumology, Children’s Hospital, Toulouse, France.; 11 San Raffaele Telethon Institute for Gene Therapy (SR-Tiget) and Pediatric Immunohematology and Bone Marrow Transplantation Unit, IRCCS San Raffaele Scientific Institute, Milan, Italy.; 12 Pediatric Hematology and Oncology, IRCCS Azienda Ospedaliero–Universitaria di Bologna, Bologna, Italy.; 13 Centro Tettamanti, Fondazione IRCCS San Gerardo dei Tintori, Monza, Italy.; 14 Department of Pediatrics, Fondazione IRCCS San Gerardo, Monza, Italy.; 15 Pediatric Hematology and Oncology Department, Hospital Sant Joan de Déu, University of Barcelona, Barcelona, Spain.; 16 Department of Neurosciences, Psychology, Drug Research and Child Health (NEUROFARBA), University of Florence, Florence, Italy.; 17 Centre of Excellence, Division of Pediatric Oncology/Hematology, Meyer Children’s Hospital IRCCS, Florence, Italy.; 18 Pediatric Oncology and Hematology Department, Miguel Servet Hospital, Zaragoza, Spain.; 19 Division of Evolution and Genomic Sciences, School of Biological Sciences, Faculty of Biology, Medicine and Health, University of Manchester, Manchester Academic Health Science Centre, Manchester, United Kingdom.; 20 “Mario Serio” Department of Experimental and Clinical Biomedical Sciences, University of Florence, Florence, Italy.; 21 Department of Pediatric Imaging, Necker Hospital for Sick Children, Paris, France.; 22 INSERM U1163, Paris, France.; 23 Laboratory of Hematology, Hospital Center of the University of Toulouse, Toulouse, France.; 24 Pediatric Unit, IRCCS Azienda Ospedaliero–Universitaria di Bologna, Bologna, Italy.; 25 Department of Medical and Surgical Sciences, Alma Mater Studiorum, University of Bologna, Bologna, Italy.; 26 Genomics Core Facility and; 27 Bioinformatic Plateform, INSERM U1163 and INSERM US24/CNRS UAR3633, Paris Cité University, Paris, France.; 28 Clinical Immunology Department, Assistance Publique Hôpitaux de Paris (AP-HP), Saint-Louis Hospital, Paris, France.; 29 Study Center for Primary Immunodeficiencies, Necker Hospital for Sick Children–AP-HP, Paris, France.; 30 Department of Hematology, CHU and Centre de Recherche de Cancérologie de Toulouse, Paul-Sabatier University, Toulouse, France.; 31 Laboratory of Immunology, Cochin Hospital, Paris, France.; 32 Garvan Institute of Medical Research, New South Wales, Australia.; 33 School of Clinical Medicine, Faculty of Medicine and Health, University of New South Wales Sydney, Sydney, Australia.; 34 Division of Pediatric Hematology and Oncology, Department of Pediatrics and Adolescent Medicine, Medical Center, Faculty of Medicine, University of Freiburg, Freiburg, Germany.; 35 Department of Pediatrics and Adolescent Medicine, University Medical Center Ulm, Ulm, Germany.; 36 Biotherapy Clinical Investigation Center, Groupe Hospitalier Universitaire Ouest, AP-HP, INSERM, Paris, France.; 37 Department of Pediatric Hematology and Oncology, Centre Hospitalo–Universitaire de Toulouse, Toulouse, France.; 38 Clinical Immunology and Primary Immunodeficiencies Unit, Pediatric Allergy and Clinical Immunology Department, Hospital Sant Joan de Déu, Barcelona, Spain.; 39 Università Vita-Salute San Raffaele, Milan, Italy.; 40 MRC Human Genetics Unit, Institute of Genetics and Cancer, University of Edinburgh, Edinburgh, United Kingdom.; 41 Centre for Molecular Medicine, Department of Medicine (Solna), Karolinska Institute, Stockholm, Sweden.; 42 School of Cancer Sciences, University of Glasgow, Glasgow, United Kingdom.; 43 Department of Pediatrics, Necker Hospital for Sick Children–AP-HP, Paris, France.; 44 Howard Hughes Medical Institute, New York, New York, USA.

**Keywords:** Autoimmunity, Immunology, Innate immunity, Monocytes

## Abstract

Patients heterozygous for germline *CBL* loss-of-function (LOF) variants can develop myeloid malignancy, autoinflammation, or both, if some or all of their leukocytes become homozygous for these variants through somatic loss of heterozygosity (LOH) via uniparental isodisomy. We observed an upregulation of the inflammatory gene expression signature in whole blood from these patients, mimicking monogenic inborn errors underlying autoinflammation. Remarkably, these patients had constitutively activated monocytes that secreted 10 to 100 times more inflammatory cytokines than those of healthy individuals and *CBL* LOF heterozygotes without LOH. *CBL*-LOH hematopoietic stem and progenitor cells (HSPCs) outgrew the other cells, accounting for the persistence of peripheral monocytes homozygous for the *CBL* LOF variant. ERK pathway activation was required for the excessive production of cytokines by both resting and stimulated *CBL*-LOF monocytes, as shown in monocytic cell lines. Finally, we found that about 1 in 10,000 individuals in the UK Biobank were heterozygous for *CBL* LOF variants and that these carriers were at high risk of hematological and inflammatory conditions.

## Abstract 2

Patients with germline *CBL* loss-of-function and somatic loss-of-heterozygosity at this locus have autoinflammation due to chronic monocyte activation through activated ERK signaling.

## Graphical abstract

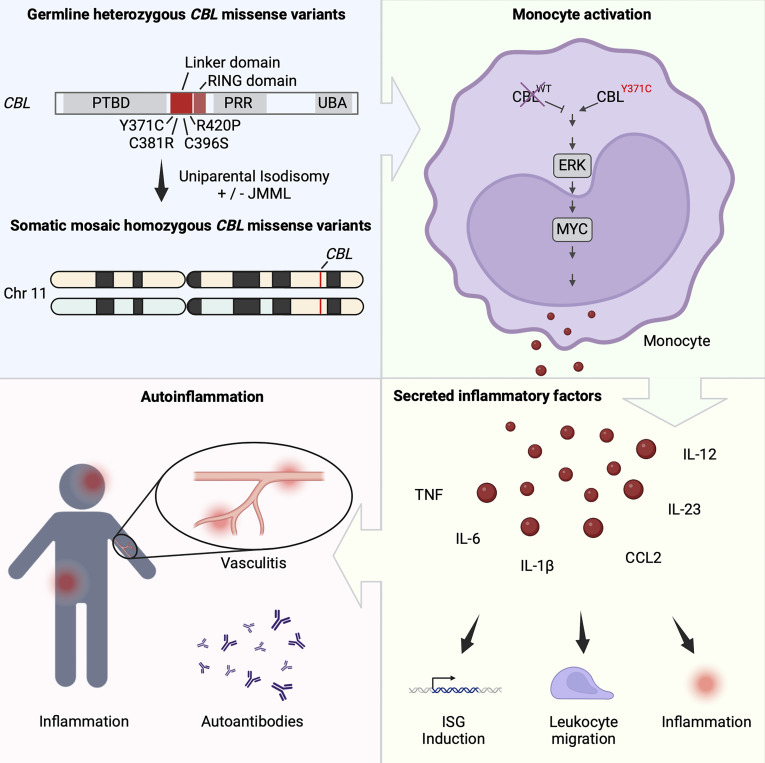

## Introduction

The downregulation of leukocyte activation prevents the excessively strong or sustained activation responsible for autoinflammation, autoimmunity, or lymphoproliferation (1–3). The human and mouse Casitas B-lineage lymphoma (CBL or c-CBL) proto-oncogenes encode an E3 ubiquitin ligase that attenuates immune and proliferative signaling by the phosphorylation-sensitive ubiquitination of receptor and non-receptor tyrosine kinases (4). CBL associates with activated tyrosine kinases via its conserved N-terminal tyrosine kinase–binding domain (TKBD), multiple residues of which are phosphorylated, including Y371 in the linker domain facilitating CBL activation (5–7). Y371 phosphorylation releases the linker domain from the TKBD of CBL, triggering interactions with the RING domain that induce the ubiquitin ligase (E3) activity of the protein (8–12). Ubiquitination of the activated tyrosine kinase then leads to its degradation, attenuating the signal it transduced. In this way, CBL downregulates several pathways, including the T cell receptor pathway via ZAP70, the B cell receptor pathway via SYK, and cytokine signaling pathways via gp130, a subunit common to many cytokine receptors (13–15). In this manner, CBL also attenuates proliferative signaling, including the EGF (16–18), FLT3L (16, 19, 20), GM-CSF (21, 22), and EPO (23) response pathways.

Germline missense variants of the RING or linker domain of human *CBL* can cause disease, particularly when E3 ubiquitination is lost but substrate-binding activity is not (*CBL* Ub^LOF^ variants) (8, 24). Germline monoallelic *CBL* Ub^LOF^ variants underlie a disorder that resembles Noonan syndrome (NS) (25, 26), a RASopathy. NS is caused by deleterious, monoallelic germline variants of the genes *NRAS*, *KRAS*, and *PTPN11* that activate the RAS pathway (27). Depending on the causal variant and affected gene, the penetrance of NS is very high or complete (28–30). NS is characterized by variable developmental and morphological defects and an increase in the risk of myeloid neoplasms. A polyclonal myeloproliferative disorder can be observed in NS neonates or infants. It can be distinguished from juvenile monomyelocytic leukemia (JMML) by clinical and hematological features, e.g., it frequently has a self-limiting course (27, 31). NS and JMML often occur together in patients with deleterious variants activating the RAS/ERK pathway; these variants are very rare in the general population (27). More than a dozen patients with NS-like disease have been reported to carry such *CBL* Ub^LOF^ variants, and these germline *CBL* variants account for about 1% of patients diagnosed with NS or Noonan-like disease (25, 32–35). Given that the incidence of NS is about 1 in 1,000–2,500 births (35), that of NS due to *CBL* Ub^LOF^ variants can be estimated at up to 10 in one million births.

Germline monoalleleic *CBL* Ub^LOF^ variants also cause JMML. The underlying mechanism involves somatic loss of the wild-type (WT) *CBL* allele via uniparental isodisomy (36). Germline *CBL* Ub^LOF^ variants with somatic loss of heterozygosity (LOH) are responsible for about 10%–15% of JMML cases (37). The annual incidence of JMML is 0.6–1.2 cases per million children under 15 years of age (38, 39); the cumulative incidence of JMML up to the age of 15 years is, therefore, up to 18 cases per million. *CBL* Ub^LOF^ variants may therefore account for up to 2.7 cases per million cases of JMML. Hematopoietic progenitors or stem cells somatically homozygous for *CBL* Ub^LOF^ variants (*CBL*-LOH) clonally outgrow the germline heterozygous bone marrow cells, resulting in a variant allele frequency (VAF) close to 100% in peripheral blood (26). *CBL*-LOH JMML is generally less aggressive clinically than JMML driven by other RAS pathway genes (*NRAS*, *KRAS*, *PTPN11*, and *NF1*), often resolving spontaneously (26). Surprisingly, after complete clinical and hematological remission of *CBL*-LOH leukemia, the VAF of the *CBL* Ub^LOF^ variant remains very high (>90%) in most or all leukocyte subsets (26, 40, 41).

Remarkably, entirely somatic homozygosity or, more rarely, somatic heterozygosity for *CBL* Ub^LOF^ variants underlies a substantial proportion of other types of myeloid leukemia (42), including approximately 10% of cases of chronic monomyelocytic leukemia (CMML) and approximately 9% of cases of acute myeloid leukemia (AML) (32, 43). Most somatic *CBL* variants are missense Ub^LOF^ variants occurring in the homozygous state (43–45). The incidence of heterozygosity for somatic variants involving the deletion of exon 8 or exons 8 and 9 is low (43–45). Heterozygosity for such deletions has been found only in AML, in combination with oncogenic variants affecting other genes, and may not therefore be sufficient to transform cells in vivo (45). By contrast, lymphoid neoplasms are extremely rare in humans with *CBL* variants, and the role of CBL in their transformation is unclear (32). Since the discovery of transforming *CBL* variants in human neoplasms, *CBL* has been viewed as a proto-oncogene (43). Hematopoietic stem cells from *Cbl^–/–^* mice have enhanced responses to pro-proliferative cytokines (46), which are further enhanced by the expression of *CBL* Ub^LOF^ variants (47). No such effect is observed when *CBL* Ub^LOF^ variants are expressed in hematopoietic stem cells from WT mice (47), a situation reminiscent of that in humans heterozygous for germline *CBL* Ub^LOF^ variants, who do not develop leukemia until LOH occurs. Thus, CBL combines the properties of a tumor suppressor (loss of ubiquitination activity) with those of a proto-oncogene (recruitment and clustering of signaling components) (24, 43, 47).

Intriguingly, patients with germline *CBL* Ub^LOF^ variants and somatic LOH displaying spontaneous JMML regression may develop autoinflammation, particularly vasculitis, either during JMML or later in life (26, 48–51). The reported frequency of autoinflammation during or after JMML in cohorts of *CBL*-LOH JMML patients is 15%–20% (26, 51). As *CBL* LOH also occurs independently of JMML (41, 42, 52, 53), the real incidence of *CBL*-driven autoinflammation is unknown. Such inflammatory disease has not been reported in patients who have undergone hematopoietic stem cell transplantation (HSCT) and achieved full donor chimerism (26, 48). Moreover, a complete reversal of vasculitis and JMML was reported in one patient with somatic *CBL* LOH after the achievement of full donor chimerism following HSCT (49). Consistent with these findings, autoimmune and autoinflammatory phenotypes occur in 30% of CMML patients before HSCT (54, 55), including those homozygous for *CBL* Ub^LOF^ variants (55). Together, these observations suggest that vasculitis is driven by *CBL*-LOH leukocytes (49, 56). The cause and mechanism of autoinflammation in these patients are unknown. In this context, we hypothesized that acquired and retained homozygosity for *CBL* Ub^LOF^ variants in leukocytes underlies clinical autoinflammation.

## Results

### CBL-LOH patients with autoinflammatory and autoimmune manifestations.

We investigated the cellular and molecular basis of autoinflammation in patients with deleterious missense *CBL* variants and LOH. We studied 12 patients recruited from 9 kindreds in 4 European countries on the basis of the presence of rare (<10^–4^) missense variants of *CBL* (Table 1). The patients carried germline monoallelic *CBL* missense variants, which were de novo in P5 and P8 and inherited from one of the parents in the others. In 9 of the 12 patients, somatic LOH via uniparental isodisomy (UPD) of chromosome 11 was detected, leading to a high VAF for the *CBL* missense variants in the blood (Table 1). Four of the 9 *CBL*-LOH patients were diagnosed with leukemia (JMML: P5, P7, P8, and P9; progression to AML in P7; see detailed case report in Supplemental Methods). Three of the 9 patients had autoinflammation (P4, P7, and P9). Specifically, P4, who was 24 years old at the time of the study, experienced episodes of retinal vasculitis, urticaria, colitis, and gastritis. P7 suffered severe cerebral vasculitis at the age of 3 years (50). HSCT led to the full resolution of AML and vasculitis in P7. P9 failed to thrive and had JMML with massive splenomegaly. He underwent splenectomy due to the hyperleukocytosis, and developed systemic arteritis (subclavian, axillary, and right brachial arteries, with the abdominal aorta, common carotid, and left brachial arteries mildly affected) 2 years later, followed by aneurysm and thrombosis. In addition, P1, P2, and P3, at the age of 11 years, presented widely distributed parenchymal lesions of the lungs on thoracic computed tomography scan, possibly indicative of inflammation. All of the patients with *CBL* LOH tested (*n* = 8) were positive for anti-nuclear antibodies, and 7 of these patients also tested positive for anti-neutrophil cytoplasmic antibodies (ANCAs), but not for antibodies against myeloperoxidase (MPO) or proteinase 3 (PR3). These patients cannot therefore be considered to have ANCA-associated vasculitis (57). Patients P4, P7, and P8 had detectable levels of other autoantibodies, such as anti–smooth muscle or anti-lupus anticoagulant antibodies (Table 1). The 3 patients without LOH (P10, P11, and P12) had no vasculitis or other features of autoinflammation. Patients P1–P5 experienced severe bacterial infections, the reason for which they were initially referred to us. These infections are currently under investigation and will be described in a subsequent manuscript. In summary, our cohort of patients with germline monoallelic *CBL* missense variants and somatic LOH presented features typical of this condition, including, in particular, vasculitis and autoantibodies.

### The patients’ CBL variants are LOF for ubiquitin ligation but do not affect substrate binding.

**Figure 1:**
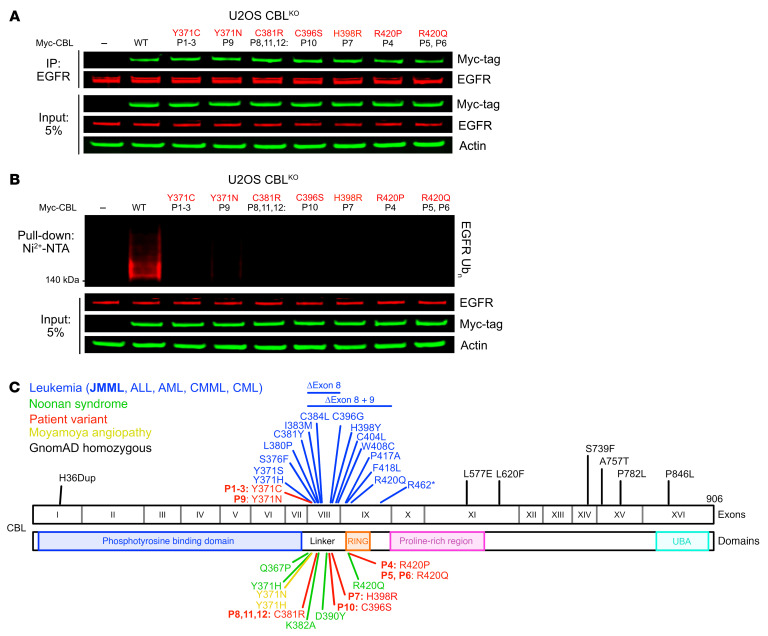
The patients’ CBL variants are Ub^LOF^ but retain substrate-binding activity. (**A**) Intact substrate binding by the CBL variants from the patients. Coimmunoprecipitation of EGFR and overexpressed Myc-tagged patient CBL variants or empty vector (EV) in CBL^KO^ U2OS cells. Anti-EGFR antibody immunoprecipitates and cell lysates were analyzed by immunoblotting. (**B**) Defective substrate ubiquitination by the CBL variants from the patients. CBL^KO^ U2OS cell lysates with overexpressed Myc-tagged patient CBL variants or EV, as indicated, along with His-Ub. (**C**) Summary of the *CBL* variants reported in patients with leukemia, NS, and moyamoya angiopathy and of the *CBL* variants present in the homozygous state in gnomAD v2.1 and the variants of the patients studied here.

We then investigated the impact of the observed CBL missense variants on CBL protein function. The N-terminal region of CBL contains the catalytic components required for the CBL-mediated downregulation of activated receptor tyrosine kinases via ubiquitination. Epidermal growth factor receptor (EGFR) is a typical substrate, and CBL functions with the E2 UBE2D2 to ubiquitinate EGFR (8, 58). CBL also interacts in a phosphorylation-dependent manner with adaptor proteins containing SH2 and SH3 domains, such as CIN85, via its C-terminus (8, 24, 59). We investigated the binding in vitro of the variant CBL proteins to two CBL substrates: ubiquitin-conjugated UBE2D2 (UBE2D2-Ub) and the EGFR peptide. Y371 phosphorylation activates the ubiquitin ligase activity of CBL (8). We therefore used CBL variants in which residue Y371 was phosphorylated (p-Y371), with the exception of CBL Y371C and CBL Y371N. We found that the variant CBL proteins and native, unphosphorylated WT CBL bound EGFR with an affinity similar to that of p-Y371 WT CBL, whereas binding to UBE2D2-Ub either occurred at a much lower level or was completely abolished (Supplemental Figure 1A and Supplemental Table 1; supplemental material available online with this article; https://doi.org/10.1172/JCI181604DS1). We then used ubiquitination reaction components that had been purified in vitro, including E1, UBE2D2, and ubiquitin (Ub), to investigate the self-ubiquitination activities of these CBL variants. Unphosphorylated WT CBL displayed little self-ubiquitination, whereas p-Y371 WT CBL was highly active (Supplemental Figure 1B), consistent with previous findings (8). The variants present in the patients were generally weakly active, if at all (Supplemental Figure 1, B–E). Only Y371N CBL was able to ubiquitinate itself at higher rates than unphosphorylated WT CBL, but this activity remained much weaker than that of p-Y371 WT CBL. These data suggest that the variants present in the patients may be dysfunctional in cells. Indeed, we found that, following overexpression in CBL-knockout (CBL^KO^) U2OS cells, the WT CBL and all the variants from the patients bound EGFR at similar rates (Figure 1A), but the EGFR ubiquitination activity of the variants was severely impaired (Figure 1B). In summary, we found that the variants present in the patients did not disturb substrate binding itself, but severely or completely impaired the substrate ubiquitination activity of CBL. These variants are, therefore, Ub^LOF^.

### Structural implications of patients’ CBL variants.

To gain insights into the effects of CBL missense variants, we mapped these variants onto the structures of CBL in both Y371-unphosphorylated and -phosphorylated states (Supplemental Figure 1, F and G). In addition, we also modeled the ubiquitin-conjugated E2 (E2-Ub) structure onto the p-Y371–CBL structure (Supplemental Figure 1H). C381, C396, and H398 are involved in coordinating the 2 zinc ions in the RING domain (Supplemental Figure 1I). Ser or Arg would abolish zinc binding and, consequently, the folding of the RING domain. In the Y371-unphosphorylated state, Y371 anchors the linker helix region (LHR) onto the TKBD by initiating both hydrophobic and hydrogen bonding interactions with the TKBD residues (Supplemental Figure 1J). Upon Y371 phosphorylation, LHR undergoes a dramatic conformational change (Supplemental Figure 1, F and G). The phosphate moiety of p-Y371 initiates hydrogen bonds with K382 and K389 in the RING domain to form a new E2-Ub binding platform (Supplemental Figure 1K). Notably, p-Y371 directly contacts Ub’s Thr9, stabilizing E2-Ub in the active conformation for catalysis. Cys or Asn prevents phosphorylation of this residue and activation of E2-Ub, thereby disrupting CBL’s E3 activity. Moreover, these variants also perturb LHR-TKBD interactions in the unphosphorylated state (Supplemental Figure 1J) and may cause LHR to release from the TKBD. R420 forms hydrogen bonds with Q42 and R72 of Ub and Q92 of E2 (Supplemental Figure 1K). This Arg, also known as the linchpin Arg, plays a critical role in stabilizing the active E2-Ub conformation for catalysis. Gln and Pro cannot support these interactions and hence hinder CBL’s E3 activity. In sum, the structural impact of the variants is consistent with loss of ubiquitin ligase activity.

### Frequent CBL variants are neutral.

We performed a literature review and identified all the available *CBL* variants reported (Figure 1C). As previously described, these *CBL* variants strongly cluster in the linker and RING domains of CBL (Figure 1C). We aimed to compare the molecular consequences of the *CBL* variants with a high frequency in the general population with those of variants reported in patients with leukemia, NS, and the Y371C variant present in P1, P2, and P3 (Figure 1C). We used the regulation of FLT3, another CBL substrate, as a molecular readout of CBL activity. We knocked out *CBL* in K562 cells, an immortalized myelogenous leukemia cell line overexpressing FLT3, and stimulated these cells with FLT3LG at various time points. In the absence of CBL, FLT3 phosphorylation was more pronounced and persisted for longer than in CBL^WT^ cells (Supplemental Figure 1L). This was expected, as CBL downregulates FLT3 protein levels, particularly for the activated, phosphorylated form. In this system, we overexpressed various CBL variants by lentiviral transduction. We confirmed that CBL^Y371C^ was LOF for the downregulation of FLT3. LOF rates were similar for the variants reported in patients with leukemia and in those with NS (Supplemental Figure 1M). Strikingly, all *CBL* missense variants present in the homozygous state in the Genome Aggregation Database (gnomAD) v2 behaved similarly to WT CBL in terms of FLT3 regulation (Supplemental Figure 1M). In summary, frequent variants of CBL are isomorphic, and our patients’ variants have the same Ub^LOF^ consequence as previously published pathogenic CBL variants.

### Excessive secretion of inflammatory cytokines by the peripheral blood mononuclear cells of patients with CBL-LOH.

**Figure 2:**
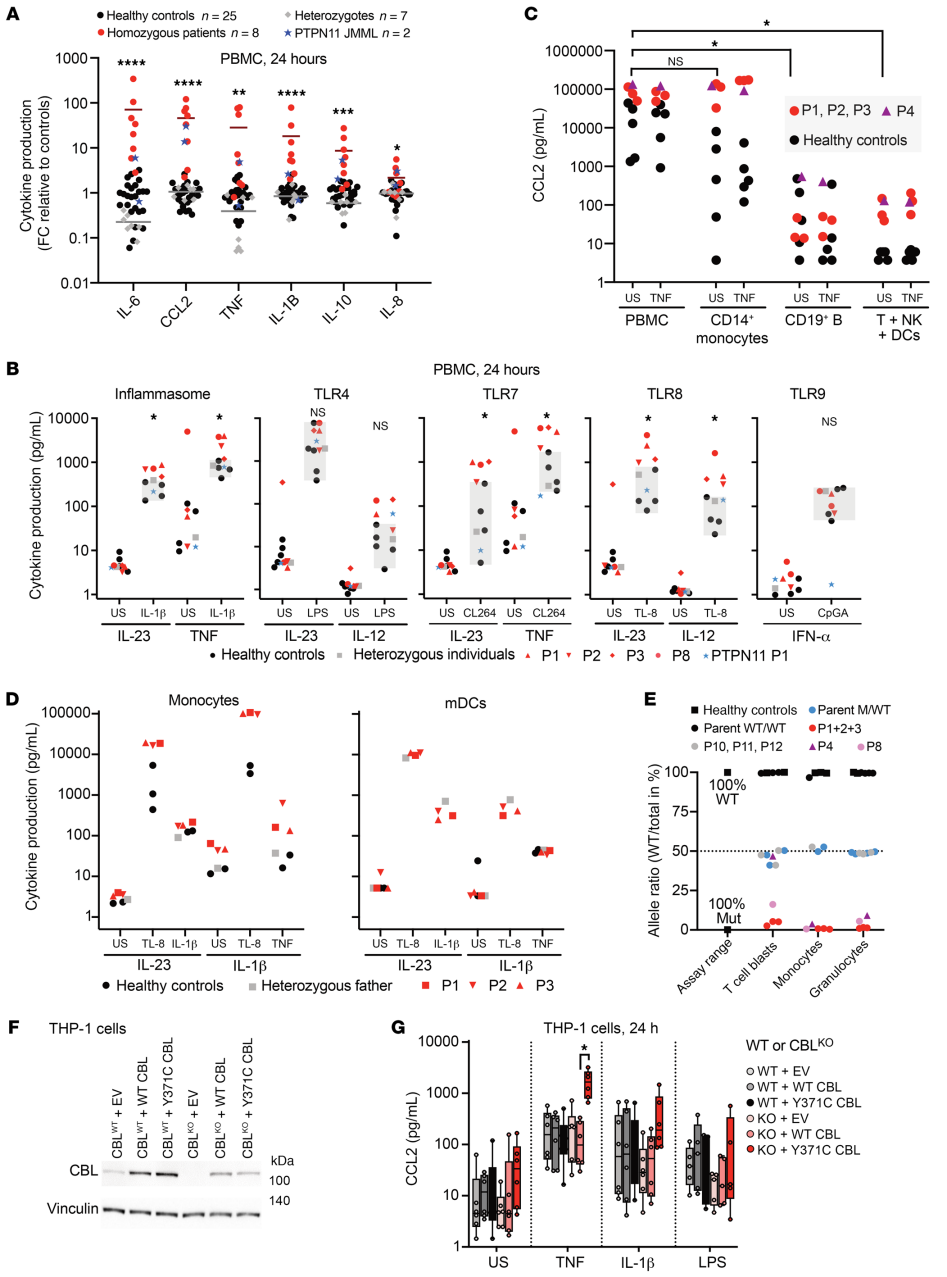
High levels of cytokine secretion by the monocytes of patients with *CBL* LOH. (**A** and **B**) The PBMCs of patients with *CBL* LOH produce excessively large amounts of inflammatory cytokines. Cytokine levels in the supernatants of PBMCs from the indicated individuals after 24 hours of culture ex vivo without stimulation (**A**) or with the indicated stimuli (**B**). Heterozygous individuals are: patients P10, P11, and P12, the father of patients P1–P3, and the grandmother, uncle, and brother of P7. Cytokine levels were assessed by bead-based ELISA. The statistical significance of differences between healthy controls and homozygous patients was assessed in multiple Mann-Whitney tests, corrected for multiple testing. *P < 0.05, **P < 0.005, ***P < 0.0005, ****P < 0.00005. FC, fold change; US, unstimulated. (**C**) PBMCs or the indicated leukocyte subsets from the indicated individuals, purified by magnetic sorting, were stimulated as indicated for 24 hours, and CCL2 levels were assessed in the supernatant. The statistical significance of differences was assessed in multiple Mann-Whitney tests, corrected for multiple testing. *P < 0.05. (**D**) Monocytes or mDCs from the indicated individuals were obtained by magnetic sorting and stimulated with the indicated agonists for 24 hours. (**E**) Variant allele frequencies of *CBL* variants in the indicated leukocyte subsets of the indicated individuals, as determined by amplicon sequencing. (**F**) Engineered THP-1 cell lines. Western blot of wild-type (CBL^WT^) and CBL-knockout (CBL^KO^) THP-1 cells generated by CRISPR/Cas9 genome editing and stably transduced with constructs: empty vector (EV), wild-type CBL (WT CBL), or Y371C-mutated CBL (Y371C CBL). (**G**) Cytokine levels in the supernatant were assessed by bead-based ELISA on the THP-1 cells shown after stimulation, as indicated, for 24 hours. *P < 0.05 by Mann-Whitney test, with correction for multiple testing.

We hypothesized that the inflammatory disease observed in *CBL-*LOH patients might be triggered by CBL-deficient leukocytes. For quantification of the proinflammatory activity of patient leukocytes, we cultured fresh peripheral blood mononuclear cells (PBMCs) from the patients, 2 patients with somatic *PTPN11* variants, healthy controls, and asymptomatic relatives for 24 hours and then quantified the cytokines secreted into the cell culture medium. The PBMCs from the patients constitutively secreted 10–100 times larger amounts of inflammatory cytokines and chemokines (Figure 2A; resolved by patient in Supplemental Figure 2A), such as IL-6, CCL2, TNF, IL-1β, IL-10, IL-8, and IL-18 (Supplemental Figure 2B), than the other cells tested. At the time of testing, P8 was on 6-mercaptopurine treatment, whereas P1, P2, P3, P4, and P6 had never had leukemia and were not on treatment. P5 and P9 displayed a spontaneous regression of JMML before sampling and were not on treatment at the time of testing. None of the patients had acute infections at the time of sampling. High plasma concentrations of IL-6, CCL2, and other cytokines were previously recorded for P7 (50), from whom PBMCs were not available for this study owing to successful HSCT. PBMCs from individuals heterozygous for *CBL* missense mutations but without somatic LOH (parents, relatives, and P10–P12) did not produce excessive amounts of these cytokines (Figure 2A). PBMCs from 2 patients with JMML driven by somatic *PTPN11* variants also produced large amounts of inflammatory cytokines, particularly CCL2, although the levels of these molecules were lower than those in most *CBL-*LOH patients (Figure 2A). These PTPN11-JMML patients were on chemotherapy (azacitidine) at the time of sampling. We profiled the high inflammatory potential of the patients’ PBMCs further, by stimulating fresh PBMCs for 24 hours with various stimuli triggering inflammatory cytokine secretion. We then analyzed the levels of the cytokines that were not present at high levels at baseline. These cells responded more strongly than control cells to triggers of the inflammasome (IL-1β), TLR7 (CL-264), and TLR8 (TL-8), but not to triggers of TLR9, and had only a mildly enhanced response to TLR4 activation (Figure 2B). These findings suggest that the proinflammatory behavior of patient PBMCs may be due to a specific cell type or a selective activating mechanism present in only certain cell types.

### CBL-LOH monocytes secrete large amounts of cytokines at baseline and upon stimulation.

We then tried to identify the leukocyte subsets responsible for this dysregulated cytokine secretion. We fractionated control and patient (P1–P4) PBMCs into CD14^+^ monocytes, CD19^+^ B cells, and the residual fraction by magnetic sorting. We found that monocytes produced most of the CCL2 (Figure 2C) and IL-6 (Supplemental Figure 2C). CD14^–^ monocytes or dendritic cells (DCs) may be responsible for the high levels of cytokine secretion by the cells of the negative fraction (Figure 2C). We also investigated the possible activation of other myeloid cells, such as granulocytes or myeloid dendritic cells (mDCs; composed of cDC1 and cDC2), in patients. Granulocytes displayed normal levels of CD62L shedding (Supplemental Figure 2D) and neutrophil extracellular trap (NET) formation (Supplemental Figure 2E) upon activation. Furthermore, purified granulocytes from P4 produced only slightly high levels of cytokines at baseline and upon stimulation (Supplemental Figure 2F), suggesting that granulocytes were not strongly dysregulated. Interestingly, patient monocytes but not mDCs produced high levels of cytokines in response to TNF or TL-8 stimulation (Figure 2D). These data suggest that monocytes are the main producers of the high levels of cytokines in patient blood. We investigated whether the monocytes of *CBL*-LOH patients were indeed homozygous for *CBL* variants at the time of our experiments. We performed quantitative amplicon sequencing (Supplemental Figure 3, A and B) on DNA from T cell populations expanded in culture by stimulation with mitogens (T cell blasts), monocytes, and granulocytes to determine the mutational burden in these subsets. Monocytes had *CBL* variant carriage rates of more than 95% in all *CBL*-LOH patients tested, as did granulocytes (Figure 2E). T cell blasts from P4 were heterozygous for the *CBL* variant, suggesting that LOH does not always affect all leukocyte subsets (Figure 2E). In summary, peripheral monocytes from patients with *CBL* LOH secrete excessive amounts of inflammatory cytokines and chemokines at baseline and upon stimulation.

### Cell-intrinsic upregulation of cytokine secretion by myeloid cells expressing CBL Ub^LOF^.

We hypothesized that homozygosity for *CBL* variants in monocytes was sufficient for activation and inflammatory cytokine secretion to be triggered in a cell-intrinsic manner. We used monomyelocytic THP-1 cells to model the genotype of the patients’ monocytes. We knocked out *CBL* by CRISPR/Cas9 gene editing and isolated a CBL^KO^ clone. We then stably transduced this clone and the CBL^WT^ THP-1 cells with WT or mutant Y371C *CBL* (Figure 2F). We focused on the Y371 residue because it acts as a mutational hotspot (Figure 1C), and because the consequences of Y371C Ub^LOF^ are representative of the effects of all the variants observed in patients (Figure 1B). As in patient monocytes, CBL^KO^ THP-1 cells overexpressing Y371C CBL, but not WT CBL, secreted large amounts of CCL2, particularly after stimulation with TNF, and, to a lesser extent, after stimulation with IL-1β (Figure 2G). These cells produced large amounts of chemokines, such as CCL2/MCP-1, I-TAC, and IP-10 (Supplemental Figure 3C). Strikingly, the overexpression of mutant CBL in WT THP-1 cells was not sufficient to trigger this phenotype, consistent with the absence of excessive cytokine secretion in heterozygous individuals. Taken together, these data indicate that homozygosity for Ub^LOF^ *CBL* variants is sufficient to confer the cell-intrinsic secretion of excessive amounts of cytokines by myeloid cells.

### Monocytosis in CBL-LOH children is driven by an increase in myeloid hematopoiesis.

**Figure 3:**
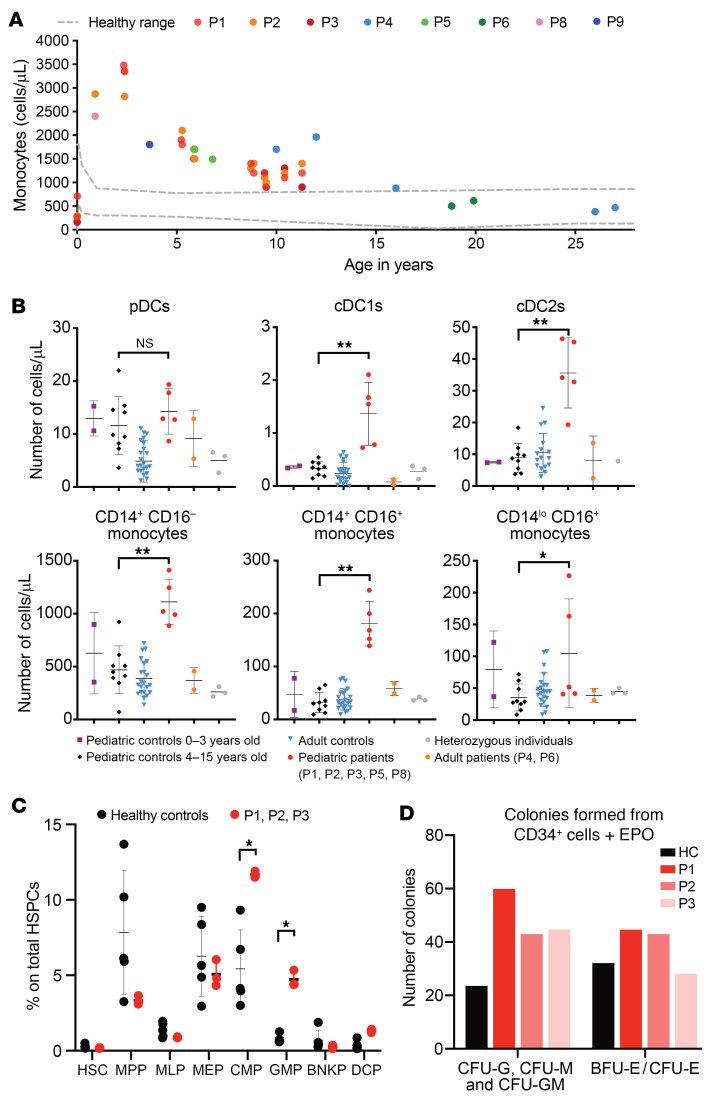
Monocytosis during childhood in *CBL*-LOH patients. (**A**) Monocyte counts per microliter of blood, as determined from clinical and research blood counts for the patients at the ages indicated. (**B**) Immunophenotyping of myeloid leukocyte subsets by mass cytometry, for the individuals indicated. *P < 0.05, **P < 0.005 by Mann-Whitney test, corrected for multiple testing. (**C**) Bone marrow phenotyping for 5 healthy controls, P1, P2, and P3. The statistical significance of differences was assessed in multiple Mann-Whitney tests, with correction for multiple testing. *P < 0.05. CMP, common myeloid progenitor; GMP, granulocytic myeloid progenitor; HSC, hematopoietic stem cell; MPP, multipotent progenitor; MLP, multi-lymphoid progenitor; MEP, megakaryocyte-erythroid progenitor; BNKP, B/NK cell progenitor; DCP, dendritic cell progenitor. (**D**) Number of colony-forming units (CFU), including CFU-GM, CFU-G, and CFU-M, and of erythroid burst-forming units (BFU-E) and erythroid colony-forming units (CFU-E) for the CD34^+^ cells of a healthy control (HC), P1, P2, and P3.

We investigated the role of monocytes in the pathogenesis of autoinflammation in more detail by characterizing the peripheral and central myeloid compartments of *CBL*-LOH patients. We aggregated complete blood count data from the 9 *CBL*-LOH patients. We observed monocytosis, particularly during the first 6 years of life, gradually regressing such that the patients had normal monocyte counts by the age of about 16 years (Figure 3A). Conversely, red blood cell, platelet, neutrophil, eosinophil, and basophil counts were within the range of healthy controls (Supplemental Figure 4, A–E). This 0illustrates that *CBL*-LOH patients often undergo a non-leukemic myeloproliferative episode early in life that is not always diagnosed as JMML. This is consistent with the current “watch and wait” standard of care for *CBL*-LOH patients diagnosed with JMML because of the high frequency of spontaneous regression in this condition (60). Importantly, the normalization of monocyte counts in peripheral blood did not coincide with a normalization of cytokine secretion. For example, at the age of 26 years, P4 displayed very high levels of cytokine secretion (Supplemental Figure 2A) despite having normal monocyte counts, as did P6 at the age of 18 years (Figure 3A). When further characterizing the myeloid compartment, we found that the pediatric *CBL*-LOH patients had high levels not only of monocytes (mainly classical and intermediate), but also of the cells of the conventional dendritic cell 1 (cDC1) and cDC2 subsets, resulting in an expansion of the myeloid DC compartment (Figure 3B). Plasmacytoid dendritic cells were present in normal numbers. We then analyzed the bone marrow of P1, P2, and P3. Bone marrow cells were morphologically normal and had a normal karyotype, and the presence of JMML was ruled out. However, using multiparametric phenotyping by flow cytometry on CD34^+^ hematopoietic stem and progenitor cells (HSPCs), we showed that the proportions of common myeloid progenitors and granulocytic myeloid progenitors were significantly increased more than 2-fold in comparison with healthy control (Figure 3C). These results were confirmed by 2 methylcellulose colony-forming unit (CFU) assays in which CD34^+^ bone marrow cells of the patients displayed an enhanced capacity to form myeloid CFU-GM, CFU-G, and CFU-M colonies compared with a healthy control in the absence of EPO (Supplemental Figure 4I) or in the presence of EPO (Figure 3D). In summary, *CBL*-LOH patients display a selective expansion of the monocyte and mDC compartments early in life.

### Transcriptional dysregulation of monocytes in CBL-LOH patients.

**Figure 4:**
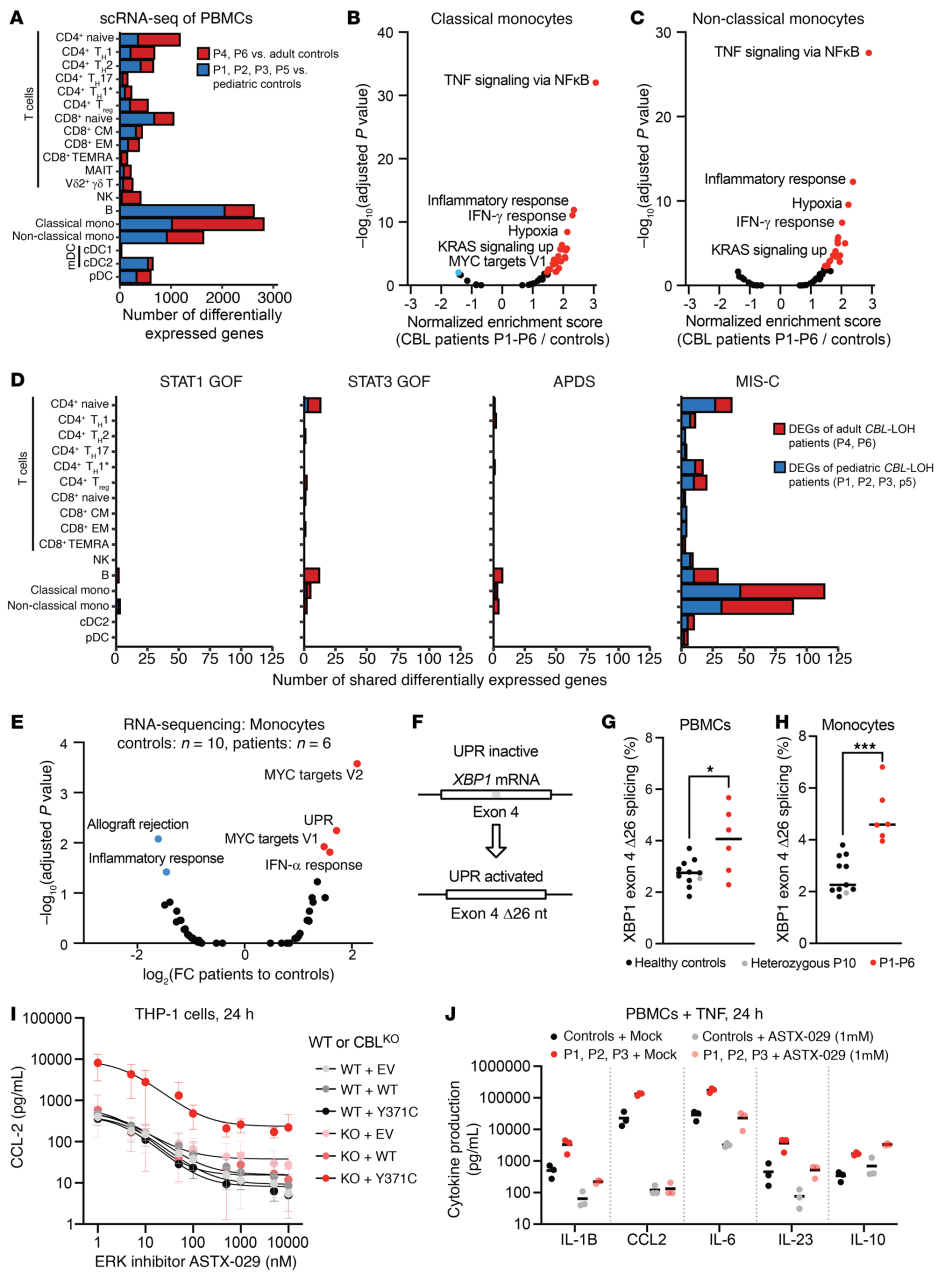
Characterization of activated monocytes in *CBL*-LOH patients. (**A**) Number of differentially expressed genes in the detected leukocyte subsets on scRNA-Seq on cryopreserved PBMCs from healthy adult (*n* = 11) and pediatric (*n* = 6) controls, and adults (*n* = 2) and children (*n* = 4) with *CBL*-LOH. (**B** and **C**) Gene set enrichment analysis of genes differentially expressed in classical (**B**) and non-classical (**C**) monocytes of *CBL*-LOH patients relative to healthy controls. (**D**) Numbers of differentially expressed genes in the detected leukocyte subsets common to *CBL*-LOH patients and patients with heterozygous gain-of-function (GOF) variants of *STAT1*, *STAT3*, and *PIK3CD* (*n* = 1, 1, and 2, respectively), and an MIS-C patient with RNase L deficiency. (**E**) Bulk RNA-Seq on healthy control (*n* = 10) and *CBL*-LOH patient monocytes after 24 hours of culture without stimulation ex vivo. Gene set enrichment analysis was performed, and the pathways for which significant enrichment was detected are shown in blue and red. (**F**) Diagram of UPR stress-induced XBP1 splicing. (**G** and **H**) Quantification of stress-dependent XBP1 splicing in PBMCs (**G**) and monocytes (**H**) from healthy controls (*n* = 10) and *CBL*-LOH patients (*n* = 6). *P < 0.05, ***P < 0.0005 by Mann-Whitney test. (**I**) Cytokine production by the indicated THP-1 cell lines following TNF stimulation with or without ASTX-029 at the indicated concentrations, including pretreatment with the inhibitor for 1 hour. Supernatants were collected after 24 hours. Dose-response curves were plotted. (**J**) Cytokine production by monocytes from patients and healthy controls stimulated with TNF with or without 1 μM ASTX-029, including pretreatment with the inhibitor for 1 hour. Supernatants were collected after 24 hours.

We characterized the phenotype of patient monocytes by performing single-cell RNA sequencing (scRNA-Seq) on cryopreserved PBMCs from P1, P2, P3, P4, P5, and P6 and comparing gene expression profiles between the cells of the patients and those of adult and pediatric controls. Classical monocytes, non-classical monocytes, and B cells were the leukocyte subsets with the largest numbers of differentially expressed genes (DEGs) relative to age-matched controls (Figure 4A). We are currently assessing the impact of the *CBL* Ub^LOF^ variants in B cells, and this work will be dealt with in another manuscript focusing on the bacterial infections of P1–P5. Gene set enrichment analysis on classical and non-classical monocyte transcriptomes revealed an enhancement of the expression of pathways relating to cytokine production (inflammatory response), cytokine signaling (TNF signaling, IFN-γ response), and ERK pathway signaling (KRAS, MYC) (Figure 4, B and C). We then compared the DEGs of adult (P4, P6) and pediatric (P1–P3, P5) *CBL*-LOH patients with the DEGs identified in other patients with monogenic autoimmune or autoinflammatory diseases. The patients tested had gain-of-function (GOF) variants of STAT1 (*n* = 1), STAT3 (*n* = 1), or PIK3CD (activated PI3K delta syndrome, *n* = 2), or RNase L LOF (multisystem inflammatory syndrome in children [MIS-C], *n* = 1) variants. A substantial overlap of DEGs was observed only with the MIS-C patient (Figure 4D).

MIS-C is an autoinflammatory condition associated with SARS-CoV-2 infection (61). Homozygous LOF variants of genes of the OAS/RNase L pathway underlie MIS-C through uncontrolled monocyte activation in response to exposure to nucleic acids derived from SARS-CoV-2 (62). We then investigated the pathways potentially driving this ectopic activation in monocytes. We performed bulk RNA-Seq on monocytes from healthy controls (*n* = 10) and *CBL*-LOH patients (*n* = 6) after 24 hours of culture ex vivo. In these conditions, fewer pathways were dysregulated, possibly because of a lack of interaction between monocytes and other leukocytes. We observed an upregulation of the transcription of MYC targets in particular (Figure 4E), consistent with ERK pathway activation. Another pathway found to be dysregulated in monocytes was the unfolded protein response (UPR). We confirmed that the UPR was activated in the monocytes of the patients, by analyzing the rate of UPR-sensitive XBP1 splicing (63) (Figure 4F) in patient monocytes and PBMCs with our RNA-Seq data and STAR aligner (https://github.com/alexdobin/STAR). Monocytes and, to a lesser extent, PBMCs from the patients displayed significantly higher rates of XBP1 splicing (Figure 4, G and H). UPR upregulation has been implicated in VEXAS syndrome, an autoinflammatory syndrome driven by somatic UBA1 LOF variants (64). Thus, the monocytes of *CBL*-LOH patients display transcriptional activation and an activation of ERK and UPR signaling, and resemble the monocytes of patients with other inflammatory diseases, such as VEXAS and MIS-C.

### RAS/ERK/MYC pathway activation is required for the CBL-mediated activation of myeloid cells.

We then hypothesized that the activation of the RAS/ERK/MYC pathway observed in patient monocytes might drive the secretory phenotype of these cells. Consistent with our findings for primary peripheral monocytes, we observed high levels of ERK1/2 phosphorylation in CBL^KO^ THP-1 cells expressing mutant CBL (Supplemental Figure 4J), consistent with homozygosity for *CBL* variants dysregulating the RAS pathway. This ERK activation was further enhanced by treatment with TNF or LPS (Supplemental Figure 4J). Inhibition of the ERK1/2 pathway with the small-molecule inhibitor ASTX-029 abolished ERK phosphorylation in WT THP-1 cells (Supplemental Figure 4J), whereas CBL^KO^ THP-1 cells overexpressing Y371C CBL were partially refractory to this inhibition. Consistently, the excessive CCL2 secretion of Y371C-expressing THP-1 cells was strongly decreased, but not completely abolished, by ERK inhibition (Figure 4I), whereas mTOR inhibition with rapamycin had no effect on the secretion of this cytokine (Supplemental Figure 4K). We also treated monocytes from P1, P2, and P3 with this ERK inhibitor and observed a marked decrease in inflammatory cytokine secretion (Figure 4J). IL-10 secretion was unaffected by this treatment, consistent with its role as a secondary, antiinflammatory response of T cells to the inflammatory environment (65). The activation of and dependence on ERK signaling are consistent with the high levels of inflammation observed in mice with *KRAS* mutations, leukemic monocytes from patients with *KRAS* mutations (66), inflammatory disease in patients with CMML (which is frequently RAS driven), and our data on PBMCs from PTPN11-JMML patients (Figure 2A). Thus, ERK activation is necessary and sufficient to induce inflammatory cytokine production by monocytes.

### Excessive inflammatory cytokine secretion triggers inflammation in vivo and in vitro.

**Figure 5:**
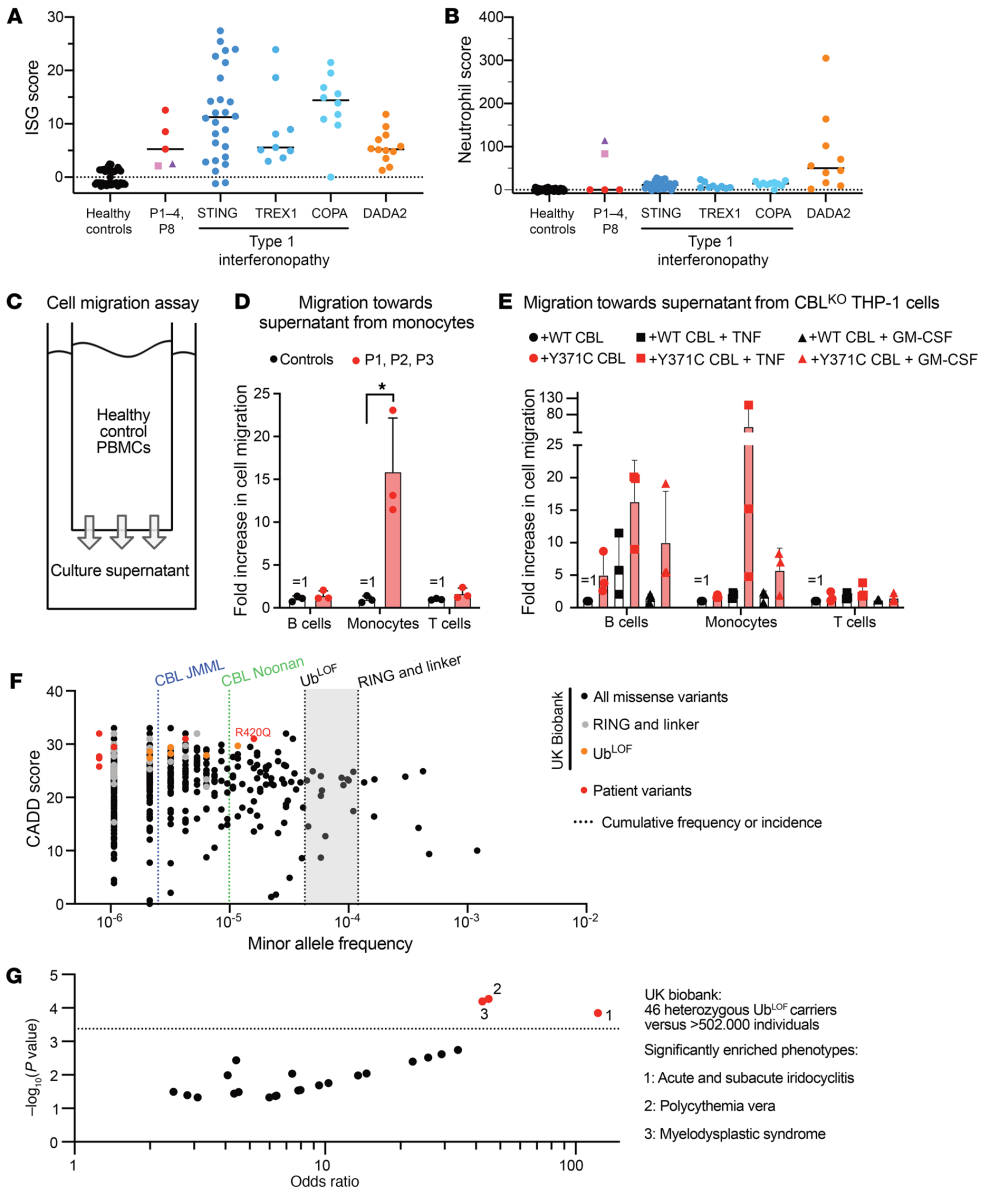
Inflammation in vivo, ex vivo, and in vitro in *CBL*-LOH patients and *CBL*-mutated THP-1 cells. (**A**) Relative expression levels for the 24 interferon-stimulated genes (ISGs) comprising the ISG score in patients P1, P2, P3, P4, and P8 relative to 27 controls. P1–P3, red; P4, purple; P8, pink. (**B**) Neutrophil scores for 27 healthy controls, the patients, previously reported individuals with type 1 interferonopathy with mutations of the indicated genes, and individuals with DADA2. P1–P3, red; P4, purple; P8, pink. (**C**) Experimental setup for PBMC Transwell migration assays. (**D**) Transwell migration of healthy control PBMCs toward cell culture supernatants from the monocytes of healthy controls or patients (P1, P2, and P3). The statistical significance of differences was assessed in a Mann-Whitney test. *P < 0.05. (**E**) Transwell migration of healthy control PBMCs toward supernatants from cultures of the indicated THP-1 cell lines after pretreatment with TNF. (**F**) Population genetics of *CBL* and CBL-driven disease. Combined annotation-dependent depletion (CADD)–minor allele frequency (MAF) plot of non-synonymous variants of CBL found in the UK Biobank genetic database (black). Patient variants, red; known Ub^LOF^ variants, orange; missense variants in the RING and linker domains, gray. The cumulative frequencies of these groups of variants are indicated. The incidence of CBL-driven NS and JMML is shown. (**G**) Phenotypes for which significant enrichment was detected in 46 carriers of Ub^LOF^ *CBL* variants in the UK Biobank relative to the 502,365 individuals of the database.

We sequenced whole-blood RNA isolated by direct lysis in PAXgene tubes (PreAnalytiX), to rule out the possibility of excessive inflammatory cytokine being an ex vivo artifact and to substantiate the inflammatory phenotype of the patient. We detected a strong inflammatory signal with NanoString probes for 24 interferon-stimulated genes (ISGs) and 6 neutrophil-associated genes (Figure 5, A and B). We also compared the cumulative ISG scores (67) of our patients with published data for patients with type 1 interferonopathies (T1Is) (67, 68) and deficiency of adenosine deaminase 2 (DADA2) (69, 70). In this semiquantitative assay, P1, P2, and P3 had a strong ISG signature, like that of T1I patients, whereas P4 and P8 did not (Figure 5A). Conversely, P4 and P8 displayed a strong neutrophil-associated gene expression signature, which is not normally observed in T1I patients but is more frequent in DADA2 patients (Figure 5B). These findings attest to the presence of cytokine-mediated inflammation in vivo. Consistent with the high levels of cytokine secretion ex vivo (Figure 2A), the *CBL*-LOH patients presented complete penetrance for molecular and cellular phenotypes of autoinflammation but incomplete clinical penetrance at their most recent follow-up visits. Our scRNA-Seq data for P1–P6 showed that all leukocyte subsets essentially displayed signatures of high levels of NF-κB, IFN, and cytokine signaling (Supplemental Figure 5A). We hypothesized that secreted factors trigger this inflammatory state in the patients’ blood. Supernatants from patient PBMCs or monocytes induced the expression of inflammatory cytokines robustly, and that of ISGs more variably, in control PBMCs (Supplemental Figure 5B). In addition to inflammatory signaling, another major step in the pathogenesis of vasculitis is the recruitment of leukocytes (particularly monocytes and neutrophils) to the site of inflammation (71). We first assessed the migration of healthy control T cells, B cells, and monocytes toward the culture supernatants of patient monocytes in a Transwell migration assay (Figure 5C). These supernatants contained high levels of several proinflammatory chemokines (Supplemental Figure 5C). Rates of monocyte migration toward patient monocyte supernatants were more than 10 times higher than those toward control supernatants (Figure 5D). Rates of granulocyte (mostly neutrophils) migration toward these supernatants were also moderately higher than those toward control supernatants (Supplemental Figure 5, D and E). Finally, we investigated whether mutant CBL expression was sufficient to elicit the secretion of molecules capable of triggering these migratory and inflammatory responses. Supernatants from THP-1 CBL^Y371C^ cells triggered high levels of monocyte and B cell migration in vitro (Figure 5E). Thus, *CBL* mutation in an isogenic cell line is sufficient to drive the secretion of chemokines recruiting monocytes. In summary, the cells of patients with *CBL* LOH have inflammatory signatures both in vivo and ex vivo. Factors secreted by the patients’ monocytes trigger this phenotype and promote autoinflammation and leukocyte migration.

### Ub^LOF^ variants occur in 1 in 10,000 people in the general population.

We investigated the population genetics of human CBL, focusing on individuals from the general population carrying Ub^LOF^ CBL variants. The biallelic (Figure 1C) or monoallelic (Supplemental Figure 6A) missense variants of CBL detected in the approximately 140,000 exomes and genomes of the gnomAD v2.1.1 database and the approximately 500,000 exomes and genomes of the UK Biobank database were not significantly less frequent in the linker and RING domains than in the rest of the protein. This suggests that the negative selection acting on the linker and RING domains of CBL may be no stronger than that acting on the other domains of the CBL protein. Consistent with this observation, the consensus-based measure of negative selection (CONES) (72) score of CBL was found to be –0.64, and the probability of intolerance to haploinsufficiency (pLI) was 0. This gene is, thus, subject to moderate negative selection. Missense variants in the linker and RING domains have a cumulative minor allele frequency (MAF) of 2 × 10^–4^ (Figure 5F and Supplemental Figure 6B). We rendered the analysis more conservative by considering only missense variants in the RING and linker domains that had already been reported in patients or tested and shown to be Ub^LOF^ (Figure 1C). The most frequent Ub^LOF^ variant was R420Q (MAF = 1.6 × 10^–5^), with 15 heterozygous carriers detected in the UK Biobank database. No Ub^LOF^ variants were found in the homozygous state in these databases. Surprisingly, these Ub^LOF^ variants had a cumulative MAF of 5 × 10^–5^, which implies that they are carried by approximately 10 in 100,000 people (Figure 1D and Supplemental Figure 2B). Importantly, this is the most conservative measurement. Currently unknown Ub^LOF^ variants may also be present in the general population. We can therefore conclude that Ub^LOF^ variants of CBL are present in at least 100 and up to 400 individuals per million in the general population. Their prevalence may be higher or lower in specific populations.

### Heterozygous Ub^LOF^ CBL variants confer a high risk of hematological and autoinflammatory disease.

Based on prevalence estimates, *CBL*-driven NS is about 10 times less frequent, and *CBL*-driven JMML is about 40 times less frequent, than the carriage of known deleterious variants of *CBL*. This large difference in frequencies may be explained by a low frequency of LOH in heterozygous carriers of Ub^LOF^ *CBL* variants and a low penetrance for NS and JMML. Consistently, several of the patients’ parents (father of P1–P3, mother of P4, mother of P6) who carry the deleterious CBL variant did not exhibit any appreciable features of NS and did not have JMML. We investigated whether carrying such Ub^LOF^ variants of *CBL* conferred risks of developing diseases other than JMML or NS. We tested for an enrichment in disease phenotypes present in at least 2 of the 46 heterozygous Ub^LOF^ carriers in the UK Biobank (*n* = 117 phenotypes). After Bonferroni correction for multiple testing, a significant enrichment in 3 disease phenotypes was detected in the variant carriers (Figure 5G and Supplemental Table 2). Two of these phenotypes were hematological: polycythemia vera (adjusted *P* value = 0.006, OR = 44) and myelodysplastic syndrome (adjusted *P* value = 0.007, OR = 42). Strikingly, neither of these myeloid hematological conditions has ever been linked to *CBL* germline variants. The third enriched phenotype was acute and subacute iridocyclitis (adjusted *P* value = 0.016, OR = 122), a type of ocular autoinflammation. In summary, carriers of Ub^LOF^ *CBL* variants are at high risk of hematological and autoinflammatory disease.

## Discussion

Our findings suggest that chronic monocyte activation underlies autoinflammation in *CBL*-LOH patients. Monocytes from patients carrying germline Ub^LOF^ *CBL* variants with somatic LOH in leukocytes had inflammatory cytokine secretion levels 10 to 100 times higher than those of monocytes from healthy controls or *CBL* Ub^LOF^ heterozygotes. No chronic monocyte activation was observed in heterozygous carriers of Ub^LOF^ *CBL* variants, who account for at least 10 in 100,000 individuals in the general population. This phenotype was reproduced in vitro by THP-1 cells engineered to express Ub^LOF^ CBL, demonstrating the cell-intrinsic nature of the effect. Monocytes homozygous for *CBL* variants persist in patients for decades. Culture supernatants from these monocytes are sufficient to trigger inflammation and leukocyte migration in vitro. We, thus, provide multiple independent lines of evidence suggesting that monocyte dysregulation triggers autoinflammation in *CBL*-LOH patients. *CBL*-LOH monocytes harbor an activated RAS/ERK/MYC signaling axis. This observation is consistent with the role of CBL in RASopathy phenotypes, such as JMML and NS-like developmental defects. The inflammatory effect of ERK activation is reminiscent of other conditions in which myeloid cells carry RAS-activating variants, such as JMML (66) and CMML (73), and of conditions in which high levels of inflammatory cytokine secretion are observed. Indeed, ERK activation is causally linked to inflammation in many instances: oncogenic KRAS G12V drives inflammation in mice and human leukemic monocytes (66), and ERK inhibition blocks inflammation due to cerebral ischemia in mice (74). We found that the inhibition of ERK signaling effectively blocked *CBL* LOH–mediated cytokine secretion ex vivo and in vitro. Additional pathways, such as the LYN-controlled PI3K/AKT signaling pathway, may also contribute to CBL-mediated inflammation, as CBL inhibits PI3K/AKT signaling via LYN degradation (75) and activating LYN variants are associated with small-vessel vasculitis (76). We also observed UPR activation in the patients’ monocytes, which may be a driver of their activation, as suggested in VEXAS syndrome (64), or may be a secondary consequence of the elevated secretory and biosynthetic activity of the cells. In summary, CBL mediates a critical, cell-intrinsic immune checkpoint in monocytes that suppresses ectopic and chronic activation and inflammatory disease.

The inflammatory cytokines secreted by patient monocytes, such as IL-6, CCL2, TNF, IL-1β, and IL-23, strongly promote inflammatory processes, such as transcriptional ISG and NF-κB induction and leukocyte migration. Monogenic errors underlying autoinflammation and vasculitis have provided evidence for the pathogenic role of these cytokines. Familial Mediterranean fever is caused by mutations of *MEFV* that increase the release of the inflammatory cytokines IL-1 and IL-18, resulting in systemic inflammation, and vasculitis in particular (77, 78). The vasculitis and autoinflammation of DADA2 patients are relieved by treatment with monoclonal antibodies directed against TNF (79–81). Deleterious *TNFAIP3* variants cause a Behçet’s disease–like autoinflammatory phenotype including retinal vasculitis (82). The A20 protein encoded by this gene downregulates NF-κB signaling, and affected patients have been shown to have high levels of inflammatory cytokines in their blood and cells (82). Biallelic hypomorphic variants of *OTULIN* underlie an autoinflammatory disorder with multiple phenotypes including vasculitis, and high levels of NF-κB, TNF, IL-1β, and IL-17 production (83) have also been observed. It is also possible that type I IFN contributes to disease in *CBL*-LOH patients. Indeed, very strong ISG expression signatures were observed in several of the patients, whereas others had normal ISG signatures. Vasculitis is a common feature of T1Is. GOF *STING* variants underlie T1I, the manifestations of which often include vasculitis (84). In summary, observations of patients with monogenic errors causing autoinflammation suggest that high levels of cytokine and IFN production may contribute to the development of vasculitis. Germline heterozygous Ub^LOF^ *CBL* variants were reported in patients with moyamoya angiopathy (48, 51, 85), a defect in the development of the neuronal vasculature, by mechanisms that remain unknown.

We estimate that 100–400 individuals per million harbor pathogenic *CBL* genotypes, whereas NS driven by germline *CBL* variants is much rarer. These observations suggest that the clinical penetrance of NS due to *CBL* Ub^LOF^ variants is markedly lower than that of NS caused by *PTPN11* variants, for example, for which penetrance is almost complete (28–30). This suggests that the pathogenic potential of *CBL* variants is significantly lower than that of *PTPN11* variants. Alternatively, CBL-driven NS-like disease may be restricted by additional unknown parameters. For example, it may require LOH in non-hematopoietic tissues or the presence of relatively frequent modifier variants in *CBL* Ub^LOF^ carriers. Consistent with this hypothesis, the cumulative incidence of CBL-driven JMML is also substantially lower than would be expected from the frequency of pathogenic genotypes. This discrepancy can probably be explained by the relatively rare occurrence of LOH at the *CBL* locus in hematopoietic cells. The mechanism by which homozygosity for *CBL* Ub^LOF^ variants underlies autoinflammation probably also operates in the autoinflammatory disease observed in patients with CMML (54) and potentially also other dysplastic or proliferative myeloid conditions driven by somatic variants of CBL or other RAS-activating lesions. Nevertheless, we found that the 1 in 10,000 *CBL* Ub^LOF^ heterozygotes in the general population were at high risk of certain myeloid hematological and inflammatory diseases. *CBL* Ub^LOF^ variants may, therefore, contribute to immune disease in two ways: as strong risk modifiers in heterozygous individuals and as monogenic drivers of disease in people with somatic LOH.

## Methods

Experimental materials and methods can be found in Supplemental Methods.

### Sex as a biological variable.

Patients in this cohort include female and male patients, and there is no clear phenotype segregation along this trait. Healthy controls of both sexes were recruited and acquired; no substantial differences along sex were observed.

### Statistics.

To assess statistical significance in this study, we generally used a *P* value cutoff of 0.05 after correction for multiple testing. Generally, we compared 2 groups: healthy controls versus *CBL*-LOH patients. The statistical significance in Figure 2, A–C and G, Figure 3, B and C, Figure 5D, Supplemental Figure 4G, and Supplemental Figure 5B was assessed by multiple Mann-Whitney tests corrected for multiple testing by 2-step step-up method (Benjamini, Krieger, and Yekutieli). The statistical significance in Figure 4, G and H, was assessed by single Mann-Whitney tests. The statistical significance in Figure 5G was assessed as follows: Enrichment *P* values were calculated in 1-tailed Fisher’s exact tests. The *P* value cutoff indicated by the dotted line was calculated by Bonferroni correction for multiple testing.

### Study approval.

Informed consent was obtained in the country of residence for each patient (Italy, France, Spain, Germany) in accordance with local regulations and with institutional review board (IRB) approval. The physicians caring for the patients completed a detailed questionnaire recording demographic data, clinical features, and biological and microbiological results, and the data were sent to J Bohlen and J Bustamante. We did not collect information about the gender or socioeconomic status of the patients. Experiments were conducted in Australia, France, Sweden, and the United States of America, in accordance with local regulations and with the approval of the IRB of The Rockefeller University (protocol JCA-0699) and INSERM (protocols C10-07 and C10-16) for the United States and France, respectively. Healthy controls were recruited in France, Spain, Italy, and the United States.

### Data availability.

All the deep-sequencing data sets are available at the Sequence Read Archive repository. scRNA-Seq data are available at PRJNA1123279. RNA-Seq data are available at PRJNA1148932.

## Author contributions

J Bohlen, IB, TV, MO, SFA, AC, LB, SS, CLF, CAAF, LS, GIR, LB, LL, CR, CM, BB, YS, TLV, DR, JR, ALN, LEB, HL, ZJ, EJC, CS, and CL conducted experiments, acquired data, and analyzed data. MM, F Barzaghi, DL, FS, FV, F Baccelli, AC, EG, MV, YADLR, F Consonni, F Conti, EM, ED, and CG collected clinical materials and information. CSM, AP, ST, IA, CBF, L Abel, ME, SYZ, VB, CLP, ES, MP, L Alsina, AA, PZ, YJC, NL, RM, DTH, JLC, J Bustamante, and J Bohlen designed research studies. J Bohlen, JLC, and J Bustamante wrote the manuscript. All authors were involved in editing the manuscript.

## Supplementary Material

Supplemental data

Unedited blot and gel images

Supplemental table 2

Supporting data values

## Figures and Tables

**Table 1:**
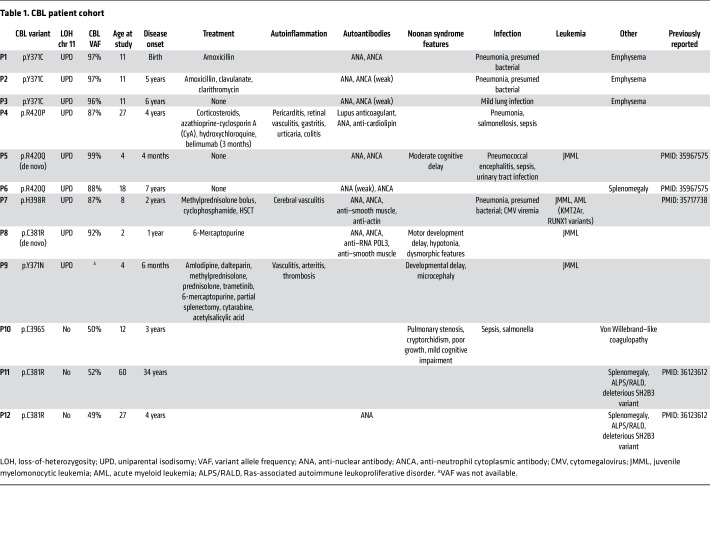
CBL patient cohort

## Version 1 10/15/2024

Electronic publication

## Version 2 10/16/2024

Corrected Dr. Bohlen's email address
