# Supplementary material for: Autoinflammation in patients with leukocytic CBL loss of heterozygosity is caused by constitutive ERK-mediated monocyte activation
Source: J Clin Invest. 2024 Oct 15;134(20):e181604. doi: 10.1172/JCI181604 (PMC11475086; doi:10.1172/JCI181604)

SUPPLEMENTAL METHODS

Extended clinical reports

P7's clinical course progressed significantly since the original publication of this case. AML progression occurred 4 months after HSCT. The clinical picture was similar to JMML relapse: spleen enlargement, monocytosis and trhombocytopenia. However, bone marrow karyogram analysis revealed a new KMT2Ar, 46, XX, t(1; 11)(q21; q23) KMT2A::EPS15. Additionally, they also found a RUNX1 mutation in the NGS analysis. So, the patient was diagnosed with an AML. Transplant immunosuppressive therapy was immediately withdrawn. The patient achieved a complete remission (CR) without additional interventions. 3 years post HSCT she is in CR.

SPR binding assays

Binding assays were performed in 150 mM NaCl, 25 mM Tris-HCl pH 7.6, 1 mM DTT, 0.005% Tween-20 at 25 °C on a Biacore T200, with a CM-5 chip (Cytiva) coupled to GST VHH (Chromotek). EGFR peptide (LQRpYSSDPTGA, 95% purity) was purchased from Generon. UbcH5B S22R C85K–Ub was produced as previously described (1). GST-tagged CBL variants encompassing residues 47-435 were captured to a level of 1,000-2,000 response units and GST alone to a level of 300-670 response units. Data are reported as the difference in signal between GST-CBL variants and GST alone. Analytes (UbcH5B S22R C85K–Ub and EGFR peptide) were serially diluted in running buffer, and binding was measured with BIAevaluation Software over the range of concentrations indicated in Figure S1A. Sensorgrams and binding curves were plotted with Prism 10 for macOS.

Cell culture

Fresh PBMCs from human blood and THP-1 cells were cultured and stimulated in RPMI medium supplemented with 5% FCS and 1% (100 IU/ml) penicillin/streptomycin. Patient-derived T-cell blasts were cultured in Immunocult medium supplemented with 1 μg/ml IL-2 and 1% (100 IU/ml) penicillin/streptomycin. HEK-293T cells were cultured in DMEM supplemented with 5% FCS and 1% (100 IU/ml) penicillin/streptomycin. All cell lines were cultured at 37 °C under a humidified atmosphere containing 5% CO_2 and 20% O_2. The human osteosarcoma cell line U2OS was obtained from ATCC and modified to generate U2OS CBL^{KO} cells. The cells were cultured in DMEM at 37 °C as a monolayer in the presence of 5% CO_2, 10% FBS, 20 mM L-glutamine, 100 IU/ml penicillin, 0.1 mg/ml streptomycin and 6 mg/l gentamycin (Invitrogen, USA). The cell line was authenticated by GenePrint 10 System Short tandem repeat profiling (Promega) and tested to confirm the absence of *Mycoplasma*. Cells were transfected with plasmids in the presence of JetPEI DNA transfection reagent (Polyplus transfection) in accordance with the manufacturer's protocol and the cells were harvested 48 h after transfection.

Plasmids, cell maintenance and transfections

The following constructs were generated in previous studies (2,3): pcDNA3.1 Myc-CBL, pcDNA3.1 Myc-CBL-Y371C, pcDNA3.1 Myc-CBL-R420Q, pcDNA3.1-12X His-Ub, pGEX4T1 GST-CBL WT (47-435), pGEX4T1 GST-CBL-Y371C (47-435), pGEX4T1 GST-CBL-R420Q (47-435), pET23d *Arabidopsis thaliana* Uba1, pRSF_1b UBE2D2 (UbcH5B), pRSF_1b-UbcH5B S22R C85K–Ub, RSF_Duet His-TEV GGS-Ub. The following constructs were generated in this study: pcDNA3.1 Myc-CBL-Y371N, pcDNA3.1 Myc-CBL-C381R, pcDNA3.1 Myc-CBL-C396S, pcDNA3.1 Myc-CBL-H398R, pcDNA3.1 Myc-CBL-R420P, pGEX4T1 GST-CBL-Y371N (47-435), pGEX4T1 GST-CBL-C381R (47-435), pGEX4T1

GST-CBL-C396S (47-435), pGEX4T1 GST-CBL-H398R (47-435), pGEX4T1 GST-CBL-R420P (47-435),

Immunoblotting and co-immunoprecipitation in U2OS cells

Before harvesting, the cells were induced by incubation with 100 ng/ml hEGF for 5 min. The cells were harvested by centrifugation and the pellet was resuspended in IP lysis buffer (50 mM Tris-HCl pH 7.5, 150 mM NaCl, 1 mM EDTA, 1% IGEPAL CA-630, 10% glycerol, 0.5 mM DTT, and cOmplete protease inhibitor cocktail). Whole-cell lysates were prepared for immunoblotting and immunoprecipitation as previously described(4). Primary antibodies were added to 1 ml freshly prepared whole-cell lysates, which were then incubated on a rotary shaker at 4 °C for 4 h. We then added 40 µl (50% slurry) Protein A Sepharose CL 4B beads to the samples, which were then incubated overnight under the same conditions. The beads were then washed three times with IP lysis buffer and the immunoprecipitated proteins were eluted in 40 µl 2X loading dye at 95 °C. Immunoblotting was performed as previously described(4) and the Odyssey CLx Imaging System (LI-COR Biosciences) was used to scan the immunoblots.

Recombinant protein purification

Human UBA1, UBE2D2 (UbcH5B) and Ub were purified as previously described(3). GST-CBL variants were inserted into the pGEX4T1 vector to generate proteins with an N-terminal glutathione S-transferase (GST)-tag followed by a thrombin cleavage site. GST-CBL variants (WT, Y371C and Y371N) were expressed in *E. coli* and purified by glutathione-affinity chromatography, followed by anion exchange and gel filtration chromatography, as previously described(5). GST-CBL p-Y371 variants (WT, C381R, C396S, H398R, R420Q and

R420P) were obtained by co-expressing pGEX4T1 GST-CBL variants and RSF_Duet His-MBP mouse Src (84-526) in *E. coli* and were purified by glutathione-affinity chromatography, followed by anion exchange and gel filtration chromatography, as previously described(5). The Bio-Rad protein assay was used to determine the concentrations of proteins other than Ub, the concentration of which was determined by measuring absorbance at 280 nm. Proteins were stored in 25 mM Tris-HCl, pH 7.6, 0.15 M NaCl and 1 mM DTT at -80 °C.

Ubiquitination assays

U2OS CBL^{KO} cells were transfected with the desired plasmids, as indicated in the figure legends, and ubiquitination assays were then performed in denaturing conditions. Cells were treated with 20 μM chloroquine for 24 h and with 100 ng/ml hEGF immediately before centrifugation. Cells were lysed in ubiquitination buffer A (UBA) (8 M urea, 0.3 M NaCl, 50 mM sodium phosphate pH 8.0, 100 μg ml^{-1} NEM), in a final volume of 750 μl. Lysates were obtained by sonication and centrifugation at 4 °C for 10 min at 20,000 x *g*. They were incubated with Dynabeads His-tag matrices (Invitrogen) overnight at 4 °C on a rotary shaker and processed as previously described(4). SDS-PAGE was used to separate the Ni^{2+}–pulldown products, and the ubiquitinated adducts were assessed by probing the immunoblots with a specific antibody. *In vitro* CBL autoubiquitination assays were performed as previously described(5). Briefly, the reactions were performed in a buffer (50 mM Tris-HCl, pH 7.6, 50 mM NaCl, 5 mM $MgCl_2$ and 5 mM ATP) containing human UBA1 (200 nM), UBE2D2 (5 μM), Ub (100 μM) and GST-CBL variant (600 nM) at room temperature. Reactions were stopped at the indicated time points by adding LDS-loading dye, subjecting the samples to SDS-PAGE with Coomassie Brilliant Blue staining and scanning the gels.

Stimulation of PBMCs, monocytes, B cells, mDCs and THP-1 cells

Blood was collected in heparin-containing tubes no more than 48 h before PBMC isolation in Lymphoprep (StemCell, 07801), in accordance with the manufacturers' instructions. PBMCs were either cryopreserved in 90% FCS, 10% DMSO or used fresh. For PBMC stimulation (fresh or cryopreserved cells), 100,000 PBMCs were used to seed each well of a round-bottomed 96-well plate, in RPMI supplemented with 10% human serum and 1% (100 IU/ml) penicillin/streptomycin. For stimulation, we added 200,000 cells per well to a 96-well V-bottom plate. Cells were stimulated for 24 hours in 100 µl RPMI and the supernatant was then collected. Cells were either left unstimulated or were stimulated with TNF (20 ng/ml), LPS (1 µg/ml), IL-1ß (25 ng/ml), TL-8 (0.1 µg/ml), CpGa (0.25 µM), or CL-264 (1 µg/ml). For ASTX-029-mediated ERK inhibition, cells were subjected to pretreatment with this inhibitor for one hour before the addition of the stimulus. The supernatant was collected 24 hours later. Cytokine secretion by PBMCs was quantified with LEGENDplexTM kits (BioLegend®) Human Inflammation Panel 1 (13-plex, #740809), HU Proinflammatory Chemokine Panel 1 (13-plex, #740985) and the Human Anti-Virus Response Panel (13-plex, #740349). Cytokine determinations were performed according to the manufacturer's protocol, except that the beads, antibody and SA-PE were diluted four-fold with the assay buffer supplied before use. Monocytes, B cells and mDCs were obtained by the magnetic sorting of total fresh PBMCs with CD14 microbeads (Miltenyi Biotec, #130-050-201), CD19 microbeads (Miltenyi Biotec, #130-050-301) and the Human Myeloid Dendritic Cell Isolation Kit (Miltenyi Biotec, # 130-094-487) according to the manufacturer's instruction. Monocytes were plated at a density of 100,000 cells per well for stimulation, whereas mDCs were plated at a density of 20,000 cells per well, with the final cytokine concentration values multiplied by five for the purposes of comparison.

Quantitative genotyping by amplicon sequencing

Whole-blood DNA was extracted with the Qiagen Blood and Tissues DNA extraction kit. DNA was obtained from leukocyte subsets by the fluorescence-activated cell sorting of subsets followed by bulk DNA extraction, except for granulocyte gDNA, which was extracted from the Lymphoprep gradient pellets obtained during PBMC purification. The variants of interest were amplified by PCR with the primers listed below.

Sequence-Direction-Variant

TGAGATGGGCTCCACATTCC-F-H398, C381, C396

CAGGCCACCCCTTGTATCAG-R-H398, C381, C396

TCTTTTGCTTCTTCTGCAGGAATC-F-R420

TCTGCTCCTTGCCTCAACAG-R-R420

GGAAACAAGTCTTCACTTTTTCTGT-F-Y371

GTGTCCACAGGGCTCAATCT-R-Y371

We used qPCR to assess the amount of DNA template in the gDNA preparations and PCR products were amplified with the smallest possible number of PCR cycles and the DreamTaq polymerase mix (Thermo, # K1071). The resulting PCR amplicons were purified and deep-sequencing libraries were prepared. Up to 100 ng of double-stranded amplicon was purified with a 1:1.6 ratio of AMPure XP SPRI magnetic beads (Beckman Coulter) and ligated with the TruSeq DNA PCR-Free Sample Preparation Kit (Illumina) according to the manufacturer’s sample preparation protocol. We then amplified 5 ng of ligated product in eight PCR cycles with KAPA HiFi HotStart ReadyMix (Roche). The amplification products were cleaned by two rounds of purification with a 1:1 ratio of AMPure XP SPRI magnetic beads (Beckman Coulter). The concentration of the final PCR products was determined by fluorimetry with the Qubit DSDNA HS assay kit (Thermo Fisher Scientific) and by capillary electrophoresis with the Fragment Analyzer HS NGS Fragment Kit (1-6000 bp) (Agilent). An equimolar pool of

libraries was prepared according to fluorimetric and capillary electrophoretic determinations and was sequenced on an Illumina NovaSeq6000 (paired-end sequencing 100+100 bases).

CD62L shedding

We incubated 100 µl heparin-treated blood for 1 hour without stimulation or with TLR agonists, IL-1R, TNF, or phorbolmyristyl acetate (PMA) at 37 °C in a humidified atmosphere containing 5% CO_2. We used 3 µg/ml LPS (TLR4, *Salmonella* Minnesota Re595 LPS, Invivogen tlrl-smlps); 0.5-5 and 20 ng/ml TNF (TNFR, Biotechne 210-TA-100), and 1 µg/ml PMA (Merck P8139-1MG). Following whole-blood activation, erythrocytes were lysed in 1.3 M NH_4Cl, 100 mM $KHCO_3$, 1 mM ethylenediaminetetra-acetic acid and washed in Roswell Park Memorial Institute 1640 medium. The contents of each tube were split into two samples, one of which was incubated for 30 minutes in dark with the fluorescein isothiocyanate–conjugated anti-human CD62L antibody, the other being incubated with the corresponding isotype control (BD Biosciences Pharmingen, 555543 and 555748). The samples were then analyzed by flow cytometry.

Stable lentiviral transduction

Lentiviral particles were produced in HEK293T cells transfected with pPAX2, pHBX2, pVSV-G and pTRIP EV or plasmids containing the *CBL* variants, with X-tremeGENE 9 (Roche, 6365779001). The medium was replaced after five hours. The supernatant was harvested 20 hours post-transfection, filtered, supplemented with 8 µg/ml protamine sulfate and added, in a 1:1 volumetric ratio, to 150,000 cells in 100 µl medium. Cells were spinoculated for 2 hours at 1200 x *g*. The cells were cultured for three to four days, assayed for transgene expression by FACS after staining for ΔNGFR, and subjected to enrichment for MACS sorting.

Generation of the U2OS, THP-1 and K562 knockout cell lines

U2OS CBL^{KO} cells were generated with the Gene Knockout Kit v2 – CBL (Synthego). U2OS cells were transfected with a pool of three CBL-targeting gRNAs in the presence of Cas9 2NLS nuclease and Lipofectamine™ Cas9 Plus Reagent with Lipofectamine™ CRISPRMAX™ Transfection Reagent, in accordance with the manufacturer's protocol. Three days later, the cells were split into smaller samples and clonal selection was performed by serial dilution. Multiple clones were genotyped, and ICE analysis (Synthego) was used to identify the percentage of clones with indels, and these clones were further verified by immunoblotting. We obtained multiple clones, and one was selected for use in the experiments performed in this study. THP-1 cells or K562 cells were transduced with pLENTI-V2 constructs encoding Cas9 and an sgRNA (AAGCTCATGGACAAGGTGAA) targeting the *CBL* gene. Cells were then selected by culture with 5 µg/ml (THP-1) or 1 µg/ml (K562) puromycin for two weeks. The KO pool was then tested for efficient knockout by western blotting, and limiting dilution methods were used for clonal expansion. Clones were screened by western blotting.

Immunoblotting

Cells were briefly washed in FCS-free DMEM or PBS and lysed in standard RIPA lysis buffer containing protease inhibitors (Roche mini EDTA-free, 1 tablet in 10 ml) and phosphatase inhibitors (2 mM sodium ortho-vanadate, Roche Phosstop 1 tablet in 10 ml, 0.1 M sodium fluoride, 0.1 M beta-glycerophosphate) and Benzonase (50 IU/ml). Lysates were clarified and protein concentration was determined in a BCA or Bradford assay. Equal amounts of protein were subjected to SDS-PAGE and the resulting bands were transferred to a nitrocellulose membrane with 0.2 µm pores. Membranes were subjected to Ponceau staining, incubated in 5% skim milk in PBST for 1 hour, briefly rinsed with PBST and then incubated overnight at 4 °C with primary antibody in 5% BSA in PBST or 5% skim milk in PBST. Membranes were then washed three times, for 15 min each, in PBST, incubated with the

secondary antibody at a dilution of 1:5,000 in 5% skim milk in PBST for 1 hour at room temperature, then washed again three times, for 15 min each, in PBST. Finally, chemiluminescence was detected with ECL reagents and a Biorad Chemidoc. The antibodies used in this study are listed in table S3. The sources of the compounds used were as follows: hEGF (Sigma–Aldrich), ATP (Fisher Bioreagents), chloroquine (Cell Signaling Technology).

NET formation

Neutrophils were isolated from whole-blood samples from healthy controls, P1, P2, P3 and P8 by density gradient centrifugation with Polymorphprep (Progen). We seeded eight-well microslides (Ibidi) with 1×10^5 neutrophils per well and incubated them at 37 °C for 15 min. PMA (100 ng/ml) was then added, and the cells were incubated for 3 h at 37 °C. The cells were fixed by incubation in 4% paraformaldehyde for 15 min, blocked and permeabilized by incubation with 5% BSA in PBS-Tween for 1 h. Cells were mounted in ProLong Gold antifade reagent containing DAPI (Thermo Fisher Scientific), and images were acquired at the Necker Institute Imaging Facility with a Leica SP8 gSTED confocal microscope (Leica). Percent NET formation was quantified by counting cells with decondensed nuclei and extracellular DAPI-positive fibers in at least five image fields selected at random.

Mass cytometry

Whole-blood mass cytometry was performed on 200 µl fresh heparinized blood from patients and controls, with a customized antibody panel, as listed in the table below, in accordance with Fluidigm recommendations. Labeled cells were subjected to dead-cell staining overnight and were then frozen and stored at − 80 °C until use. Acquisition was performed on a Helios machine (Fluidigm) and the data were analyzed with OMIQ software.

RNA sequencing analysis

RNA was isolated from PBMCs or monocytes with Quick-RNA™ kits (Zymo, R1052) after 24 hours of culture *ex vivo*. Total RNA sequencing was performed with an Illumina NovaSeq S2 flowcell (read length 100 bp) with a read depth of 30 M. All FASTQ sequences passed quality control tests and were aligned with the GRCh38 reference genome with STAR (2.6.1d), which is configured to detect splice junctions. BAM files were converted to a raw-count expression matrix with featurecount. Raw-count data were normalized with DEseq2. The ensemble ID targeting multiple genes was collapsed (average) and a final gene data matrix was used for a modular repertoire analysis as previously described (6,7) or for gene set enrichment analysis (GSEA: fgsea) with hallmark gene sets (http://www.gsea-msigdb.org/). We analyzed the splice junctions in the genomic region of interest (exons 3-5 of *XBP1*, especially the alternative splicing events within exon 4 of *XBP1*) and visualized them with Sashimi plots.

PBMC stimulation for RT-qPCR

For control PBMC stimulation with culture supernatants from patient and control PBMCs, we seeded plates with 100,000 PBMCs per well and incubated these cells with the supernatants tested, at a 1:10 dilution, for 24 hours. Total RNA was then extracted with Quick-RNA™ kits (Zymo, R1052). The High-Capacity RNA-to-cDNA™ Kit (Applied Biosystems, #4387406) was used to synthesize cDNA and target cDNAs were quantified by *Taq*man probe qPCR with the Master Mix for PCR TaqMan™ Fast Universal (2X) (Applied Biosystems, # 4366073). The following *Taq*man probes were used: IL1B - Hs00174097_m1, IL8- Hs00174103_m1, IL6- Hs00174131_m1, IFI27- Hs01086373_g1, MX1- Hs00895608_m1, IFI44L - Hs00199115_m1 and OAS1- Hs00973637_m1.

Transwell migration assay

Culture supernatants for migration analysis were generated by seeding plates with THP-1 cells at a density of 2 x 10^6 cells/ml and stimulating them as indicated. After 24 hours of incubation at 37 °C, cells were washed twice with RPMI to remove cytokines and stimulants and were then resuspended in the same volume of RPMI without stimulation. The supernatant was harvested 24 hours after medium replacement, by centrifugation and filtering of the supernatant through sterile filters with 0.2 µm pores. For migration analysis, 600 µl of cell-free THP-1 supernatant was added to each well of a 24-well plate, along with 10,000 agarose beads for the normalization of cell numbers. When a supernatant from patient monocytes was used, 6 µl culture supernatant was added to 594 µl RPMI (1:100 dilution). For the analysis of cell migration, cell culture inserts with a 5.4 µm-pore membrane at the bottom were placed on top of the supernatant and 200,000 fresh PBMCs or 20,000 granulocytes from healthy controls in 100 µl RPMI were added to the insert. Granulocytes were obtained by density centrifugation on Polymorphoprep (Proteogenix, #11146832) according to the manufacturer's instructions. Blanks consisting of RPMI medium alone rather than supernatant were used as the negative control. After 2 hours at 37 °C, the cells that had migrated were collected and stained for extracellular markers for quantification and counting by flow cytometry. The cells that had migrated and beads were counted and quantified with FlowJoTM. The number of cells migrating in the blanks was subtracted from the number of cells in the supernatant of coculture samples to determine the net number of cells that could be considered to have migrated in response to stimulation. Cell numbers were normalized against bead counts and increases in the migration of T cells ($CD3^+$), monocytes ($CD14^+$), B cells ($CD19^+$) and neutrophils ($CD11b^+$ and $CD16^+$) were determined relative to the unstimulated WT CBL control.

Single-cell RNA sequencing

PBMCs from the CBL LOH patients P1-P6, the mother of P1-P3 (CBL WT/WT), the father of P4 (CBL WT/WT), six age-matched controls, and 11 adult controls were subjected to single-cell RNA sequencing (scRNASeq) analysis as previously described(8). We investigated the mechanisms underlying the diseases caused by CBl LOH, by also including controls presenting pathological manifestations with heterozygous gain-of-function (GOF) mutations of *STAT1*, *STAT3*, and *PIK3CD* (*N*=1, 1, and 2, respectively), and an MIS-C patient with RNase L deficiency. The data for the *STAT1* GOF patient and the MIS-C patient have been published elsewhere(9,10). Pseudobulk differential expression analysis was performed as previously described(8).

Isolation of mononuclear cells from bone marrow

Bone marrow mononuclear cells (BM-CMNs) were isolated by density gradient separation on Lymphoprep (Axis-Shield, Oslo, Norway). We extracted CD34$^+$ hematopoietic progenitor cells from the BM-CMN fraction with an indirect CD34 microbead kit and a magnetic separator (Miltenyi Biotec, Bergisch Gladbach, Germany). The purity of the CD34$^+$ fraction was evaluated with a NovoCyte flow cytometer (Agilent, CA, USA).

Soft agar colony assay

The ability of CD34$^+$ cells to form CFUs and BFUs was evaluated in a clonal assay on methylcellulose (MethoCult H04535 and H04435, Stemcell Technologies, Vancouver, Canada) after culture for eight to 12 days according to the standard protocol provided by Stemcell Technologies. The medium contains the following cytokines: Recombinant human stem cell factor (SCF), Recombinant human interleukin 3 (IL-3), Recombinant human interleukin 6 (IL-6), Recombinant human erythropoietin (EPO) (for H4435 only),

Recombinant human granulocyte colony-stimulating factor (G-CSF), Recombinant human granulocyte-macrophage colony-stimulating factor (GM-CSF).

Bone-marrow phenotyping

For phenotyping, cells from patients and healthy donors were characterized with the multi-labeled panel of antibodies listed below. Cell analysis was performed with a NovoCyte or a Sony SP6800 spectral flow cytometer. The data were analyzed with FlowJo software. The following Ab panel was used for the analysis of bone-marrow cells: anti-CD19 APCCY7 (#5302218, Biolegend), anti-IgM BV421 (#562618, from BD), anti-CD45RA BV711 (#304138, Biolegend), anti-CD90 PE-Cy5 (#328112, Biolegend), anti-CD38 BV605 (#303532, Biolegend), anti-CD34 APC-Cy7 (#343514, Biolegend), anti-CD133 PE-Dazzle (#372812, Biolegend), anti-CD123 PE-Cy7 (#306010, Biolegend), anti-CD10 BV510 (#312220, Biolegend), anti-CD110 BV421 (#743955, BD) and a customized Lin panel including anti-CD2 , anti-CD3, anti-CD4, anti-CD8, anti-CD13, anti-CD14, anti-CD15, anti-CD16, anti-CD19, anti-CD20, anti-CD56, and anti-CD235a antibodies, all conjugated with PE and obtained from Miltenyi Biotec.

LEGENDS TO SUPPLEMENTAL FIGURES

Supplemental Figure 1: Functional assessment of the patients' *CBL* variants. (A) SPR analyses of glutathione-S-transferase (GST)-CBL variants and analyte binding affinities, related to Table S1. Representative sensorgrams (left) and binding curves (right) for GST-CBL variants (indicated on the left) and analytes (indicated at the top). **(B-E)** SDS-PAGE in reducing conditions, showing the self-ubiquitination of GST-CBL variants *in vitro* in the

presence of ubiquitination components, including E1, UBE2D2 and ubiquitin. The Coomassie Brilliant Blue-stained gel shows the presence of Ub, UBE2D2, GST-CBL, and ubiquitinated GST-CBL-Ub (GST-CBL-Ub_n). Asterisks indicate the E1 band. (F-H) Mapping of CBL missense variants onto the structure of CBL bound to E2 UBE2L3 (F; PDB: 1FBV), p-Y371-CBL bound to E2 UBE2D2 (G; PDB: 4A4C), and p-Y371-CBL bound to a modelled E2–Ub (H; E2–Ub from pY363-CBLB bound to E2–Ub (PDB: 3ZNI) was modelled onto the structure of p-Y371-CBL bound to E2). Structures of CBL in F-H are aligned based on the TKBD domain and shown in the same orientation. TKBD is coloured in blue, LHR in yellow, RING in orange, E2 in cyan, and Ub in green. Missense variants are represented by red spheres, and zinc ions by grey spheres. (I) Close-up view of zinc coordinating ligands in the RING domain from F. (J) Close-up view of the Y371-binding interface in F. (K) Close-up view of p-Y371 and R420 binding interfaces from H. (L) CBL^{KO} K562 cells overexpressing FLT3 display delayed FLT3 deactivation. Western blot of K562 cells stimulated with FLT3LG for the indicated periods of time. **(M)** The patients' Y371C *CBL* variant is Ub^{LOF}, as are previously published CBL variants detected in patients with myeloid leukemia or NS. Variants found in the homozygous state in gnomAD v2.1 are, however, isomorphic. Western blot of K562 CBL^{KO} cells stably transduced with the indicated CBL constructs and treated with FLT3LG for 3 hours.

Supplemental Figure 2: High levels of cytokine production by the PBMCs of patients with *CBL*-LOH. (A) The large amounts of inflammatory cytokines produced by the PBMCs of patients with *CBL*-LOH, as shown in Figure 2A, are resolved, by patient, here. Cytokine levels in the supernatants of PBMCs from the indicated individuals after 24 hours of culture without stimulation *ex vivo*. **(B)** The PBMCs of patients with CBL-LOH produce excessively large

amounts of IL-18. Cytokine levels in the supernatants of PBMCs from the indicated individuals after 24 hours of culture *ex vivo* without stimulation. **(C)** PBMCs or the indicated leukocyte subsets from the individuals shown were purified by magnetic sorting and stimulated, as indicated, for 24 hours. IL-6 levels in the supernatant were then determined. The statistical significance of differences was then assessed in Mann-Whitney tests. *$p<0.05$. **(D)** Normal rates of CD62L shedding by the patients' granulocytes. **(E)** Normal rates of NET formation by the patients' granulocytes. **(F)** Moderately high levels of cytokine production by the granulocytes of P4.

Supplemental Figure 3: Amplicon sequencing quality control and THP-1 cell characterization. **(A)** Calibration curve for amplicon sequencing for the CBL p.Y371C variant, based on biological triplicates. **(B)** Representative plot of aligned read counts for the nucleotides of the p.Y371C variant in the sample of saliva from P1. **(C)** Cytokine and chemokine levels in supernatants, as assessed by bead-based ELISA on the indicated THP-1 lines after 24 hours of stimulation, as indicated.

Supplemental Figure 4: Immunophenotyping of peripheral and central leukocytes from the patients.

(A-E) Blood cell counts of the indicated patients at the indicated age. Plots show the healthy range and patient values of red blood cells **(A)**, palelets **(B)**, neutrophils **(C)**, eosinophils **(D)** and basophils **(E)**. **(F-H)** Immunophenotyping, by mass cytometry, of neutrophils **(F)**, eosinophils **(G)** and basophils **(H)** subsets from the indicated individuals. **(I)** Formation of white myeloid cell colonies from $CD34^{+}$ cells from the bone marrow of a healthy control, P1, P2 and P3, in the absence of EPO. **(J)** High levels of cytokine production by CBL^{Y371C} THP-1 cells mediated by activated ERK signaling. The three THP-1 lines indicated were stimulated

with TNF or LPS (concentrations in the methods section) for 2 hours in the presence or absence of the ERK/MEK inhibitor ASTX-029 at a concentration of 1 µM, after preincubation for one hour with the inhibitor. A western blot was performed. **(K)** Production of cytokines by the indicated THP-1 cell lines following stimulation with TNF in the presence or absence of rapamycin at the indicated concentrations, with preincubation for one hour with the inhibitor. Supernatants were collected after 24 hours and dose-response curves were plotted.

Supplemental Figure 5: Altered immune functions and phenotypes in *CBL*-LOH patients. **(A)** Gene-set enrichment analysis of single-cell RNA-sequencing data. Pathways significantly enriched or depleted in at least four subsets are shown. **(B)** Relative levels of the indicated mRNAs in healthy control PBMCs incubated with monocyte culture supernatants from healthy controls or the indicated patients for 24 hours, as determined by RT-qPCR. The statistical significance of differences was assessed in Mann-Whitney tests. *p<0.05 **(C)** Cytokine and chemokine production by the patients' monocytes. Monocytes were obtained from the indicated individuals by magnetic sorting, incubated for 24 hours and cytokine levels in the supernatant were then determined. **(D)** Experimental setup for granulocyte trans-well migration assays. **(E)** Trans-well migration of healthy control granulocytes towards the supernatants of cultures of monocytes from healthy controls or patients (P1, P2 and P3).

Supplemental Figure 6: Population genetics of *CBL*. **(A)** Minor allele frequency of *CBL* missense variants along the length of the CBL protein sequence present in the gnomAD v2.1 and UK biobank databases. **(B)** Population genetics of CBL and CBL-driven disease. CADD-MAF plot of non-synonymous variants of CBL found in the gnomAD v2.1 database (black). The patients' variants are shown in red. Known Ub^{LOF} variants are shown in orange. Missense variants in the RING and linker domains are shown in gray. The cumulative frequencies of

these groups of variants are indicated. The incidence of CBL-driven NS and JMML is also shown.

Table S1. Equilibrium dissociation constants (K_D) for interactions between CBL (residues 47–435) variants and UBE2D2–Ub or EGFR peptide.

Immobilized GST–CBL variant	Analyte	K_D (μM)
WT	UBE2D2–Ub	41 ± 4
p-Y371	UBE2D2–Ub	0.37 ± 0.06
Y371C	UBE2D2–Ub	19 ± 1
Y371N	UBE2D2–Ub	8.3 ± 0.6
p-Y371 C381R	UBE2D2–Ub	N.M.
p-Y371 C396S	UBE2D2–Ub	N.M.
p-Y371 H398R	UBE2D2–Ub	N.M.
p-Y371 R420P	UBE2D2–Ub	9.3 ± 0.5
p-Y371 R420Q	UBE2D2–Ub	4.1 ± 0.3
WT	EGFR peptide	2.3 ± 0.1
p-Y371	EGFR peptide	1.4 ± 0.1
Y371C	EGFR peptide	1.6 ± 0.1
Y371N	EGFR peptide	1.1 ± 0.1
p-Y371 C381R	EGFR peptide	2.3 ± 0.3
p-Y371 C396S	EGFR peptide	2.4 ± 0.2
p-Y371 H398R	EGFR peptide	2.5 ± 0.3
p-Y371 R420P	EGFR peptide	1.5 ± 0.1
p-Y371 R420Q	EGFR peptide	1.4 ± 0.1

s.e.m. are indicated for n=4 replicates for UBE2D2–Ub and n=6 replicates for EGFR peptide. N.M. = not measurable. Representative sensorgrams and binding curves are shown in Figure S1A.

Table S3. Antibodies used in this study for western blotting.

Antibody	Reference	Dilution factor
FLT3	Cell SignalingTechnology, #3462S	1:1000
p-FLT3	Cell SignalingTechnology, #3464S	1:1000
CBL	Cell SignalingTechnology, #2747S	1:1000
Vinculin HRP	Santa Cruz, #sc-73614 HRP	1:5000
Lamin A/C HRP	Santa Cruz, #sc-376248 HRP	1:1000
EGFR	Merck Millipore, #06-847	1:1000 WB, 1:500 IP
Myc-tag	Cell Signaling, #2276	1:1000
Actin	Santa Cruz, #sc-47778	1:1000
ERK	Cell SignalingTechnology, #4370S	1:1000
pERK	Cell SignalingTechnology, #9106S	1:1000

Table S4. Antibodies used in this study for mass cytometry.

089Y	CD45	HI30	3089003B	Fluidigm
116Cd	CD66b	QA17A51	396902	Biolegend
141Pr	CCR6	G034E3	3141003A	Fluidigm
142Nd	CD19	HIB19	3142001B	Fluidigm
143Nd	CD127	A019D5	3143012B	Fluidigm
144Nd	CD38	HIT2	3144014B	Fluidigm
145Nd	CD31	WM59	3145004B	Fluidigm
146Nd	IgD	IA6-2	3146005B	Fluidigm
147Sm	CD11c	Bu15	3147008B	Fluidigm
148Nd	CD20	2H7	302302	Biolegend
149Sm	CD25	2A3	3149010B	Fluidigm
150Nd	NKVFS1	NKVFS1	MCA2243GA	Bio Rad
150Nd	KIR3DL1L2	REA970	130-126-489	Miltenyi Biotec
151Eu	CD123	6H6	3151001B	Fluidigm
152Sm	TCR-γδ	11F2	3152008B	Fluidigm
153Eu	Va7.2	3C10	3153024B	Fluidigm
154Sm	CD3	UCHT1	3154003B	Fluidigm
155Gd	CD45RA	HI100	3155011B	Fluidigm
156Gd	CCR10	REA326	130-122-317	Miltenyi Biotec
158Gd	CD27	L128	3158010B	Fluidigm
159Tb	CD1c	L161	331502	Biolegend
160Gd	CD14	M5E2	3160001B	Fluidigm
161Dy	CLEC9A	8F9	3161018B	Fluidigm
162Dy	CD21	REA940	130-124-315	Miltenyi Biotec
163Dy	CXCR3	G025H7	3163004B	Fluidigm
164Dy	CD161	HP-3G10	3164009B	Fluidigm
165Ho	NKG2C	REA205	130-122-278	Miltenyi Biotec
166Er	CD24	ML5	3166007B	Fluidigm
167Er	CCR7	G043H7	3167009A	Fluidigm
168Er	CD8	SK1	3168002B	Fluidigm
169Tm	NKG2A	Z199	3169013B	Fluidigm
170Er	iNKT	6B11	3170015B	Fluidigm
171Yb	CXCR5	RF8B2	3171014B	Fluidigm
172Yb	CD57	HNK-1	359602	Biolegend
173Yb	HLA-DR	L243	3173005B	Fluidigm
174Yb	CD4	RPA-T4	300502	Biolegend
175Lu	CCR4	L291H4	3175035A	Fluidigm
176Yb	CD56	NCAM16.2	3176008B	Fluidigm
209Bi	CD16	3G8	3209002B	Fluidigm

Supplemental References:

1. Dou, H., Buetow, L., Sibbet, G.J., Cameron, K. and Huang, D.T. (2013) Essentiality of a non-RING element in priming donor ubiquitin for catalysis by a monomeric E3. *Nat Struct Mol Biol*, **20**, 982-986.
2. Buetow, L., Tria, G., Ahmed, S.F., Hock, A., Dou, H., Sibbet, G.J., Svergun, D.I. and Huang, D.T. (2016) Casitas B-lineage lymphoma linker helix mutations found in myeloproliferative neoplasms affect conformation. *BMC Biol*, **14**, 76.
3. Magnussen, H.M., Ahmed, S.F., Sibbet, G.J., Hristova, V.A., Nomura, K., Hock, A.K., Archibald, L.J., Jamieson, A.G., Fushman, D., Vousden, K.H. *et al.* (2020) Structural basis for DNA damage-induced phosphoregulation of MDM2 RING domain. *Nat Commun*, **11**, 2094.
4. Ahmed, S.F., Buetow, L., Gabrielsen, M., Lilla, S., Sibbet, G.J., Sumpton, D., Zanivan, S., Hedley, A., Clark, W. and Huang, D.T. (2021) E3 ligase-inactivation rewires CBL interactome to elicit oncogenesis by hijacking RTK-CBL-CIN85 axis. *Oncogene*, **40**, 2149-2164.
5. Dou, H., Buetow, L., Hock, A., Sibbet, G.J., Vousden, K.H. and Huang, D.T. (2012) Structural basis for autoinhibition and phosphorylation-dependent activation of c-Cbl. *Nat Struct Mol Biol*, **19**, 184-192.
6. Altman, M.C., Rinchai, D., Baldwin, N., Toufiq, M., Whalen, E., Garand, M., Syed Ahamed Kabeer, B., Alfaki, M., Presnell, S.R., Khaenam, P. *et al.* (2021) Development of a fixed module repertoire for the analysis and interpretation of blood transcriptome data. *Nat Commun*, **12**, 4385.
7. Rinchai, D., Roelands, J., Toufiq, M., Hendrickx, W., Altman, M.C., Bedognetti, D. and Chaussabel, D. (2021) BloodGen3Module: Blood transcriptional module repertoire analysis and visualization using R. *Bioinformatics*.
8. Ogishi, M., Arias, A.A., Yang, R., Han, J.E., Zhang, P., Rinchai, D., Halpern, J., Mulwa, J., Keating, N., Chrabieh, M. *et al.* (2022) Impaired IL-23-dependent induction of IFN-gamma underlies mycobacterial disease in patients with inherited TYK2 deficiency. *J Exp Med*, **219**.
9. Moriya, K., Nakano, T., Honda, Y., Tsumura, M., Ogishi, M., Sonoda, M., Nishitani-Isa, M., Uchida, T., Hbibi, M., Mizoguchi, Y. *et al.* (2023) Human RELA dominant-negative mutations underlie type I interferonopathy with autoinflammation and autoimmunity. *J Exp Med*, **220**.
10. Lee, D., Le Pen, J., Yatim, A., Dong, B., Aquino, Y., Ogishi, M., Pescarmona, R., Talouarn, E., Rinchai, D., Zhang, P. *et al.* (2023) Inborn errors of OAS-RNase L in SARS-CoV-2-related multisystem inflammatory syndrome in children. *Science*, **379**, eabo3627.

Supplemental Figure 1

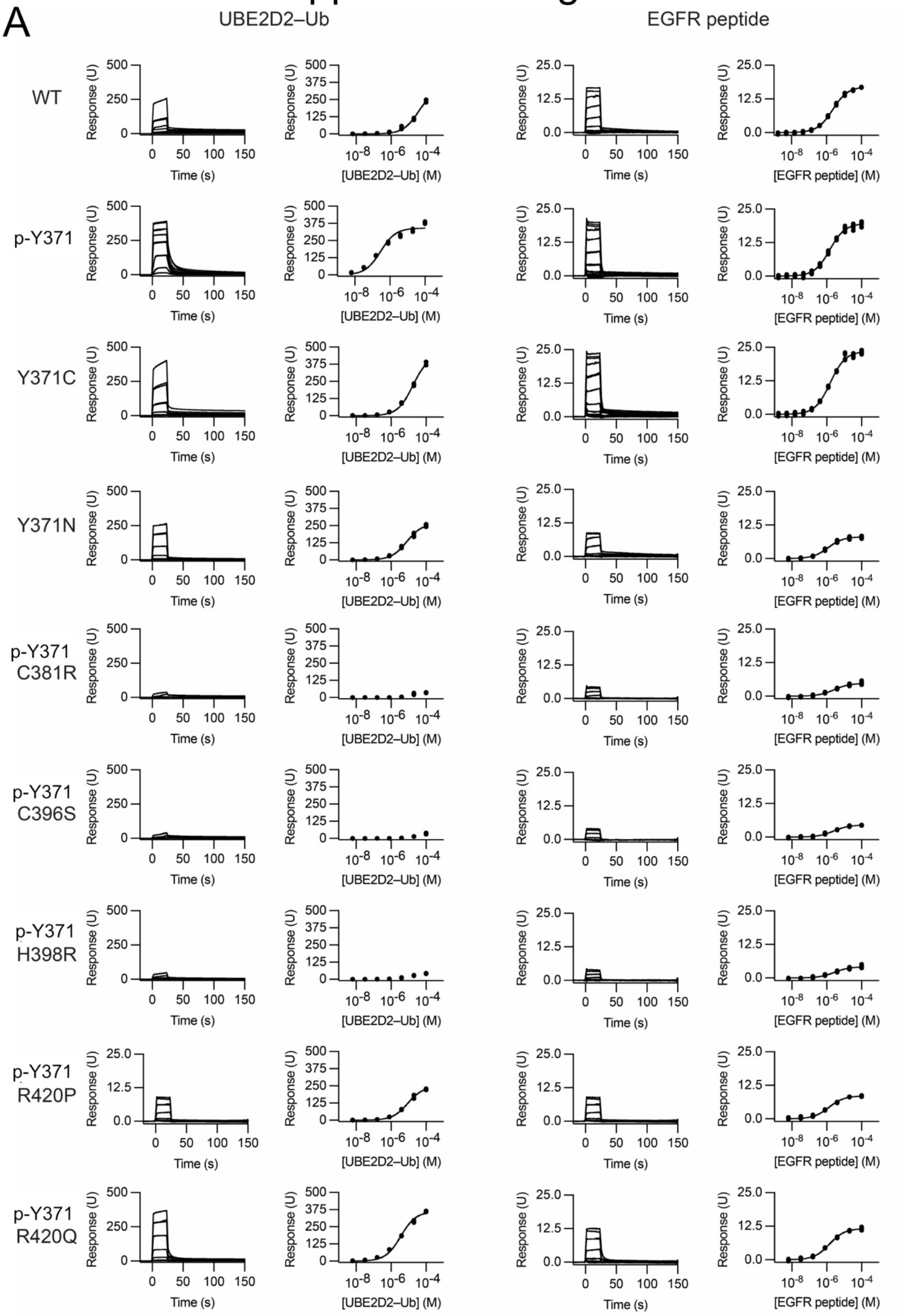

Supplemental Figure 1 continued

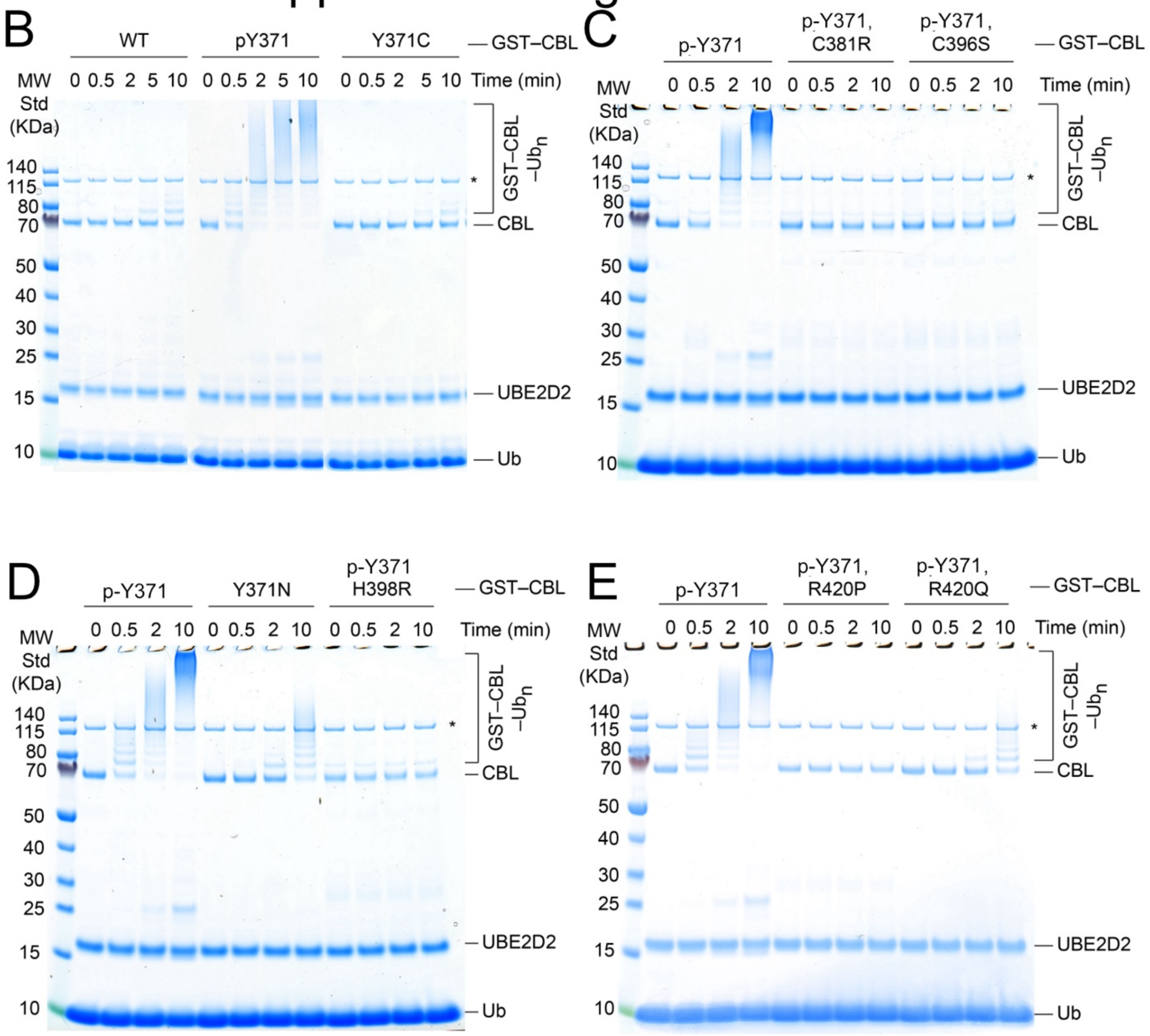

Supplemental Figure 1 continued 2

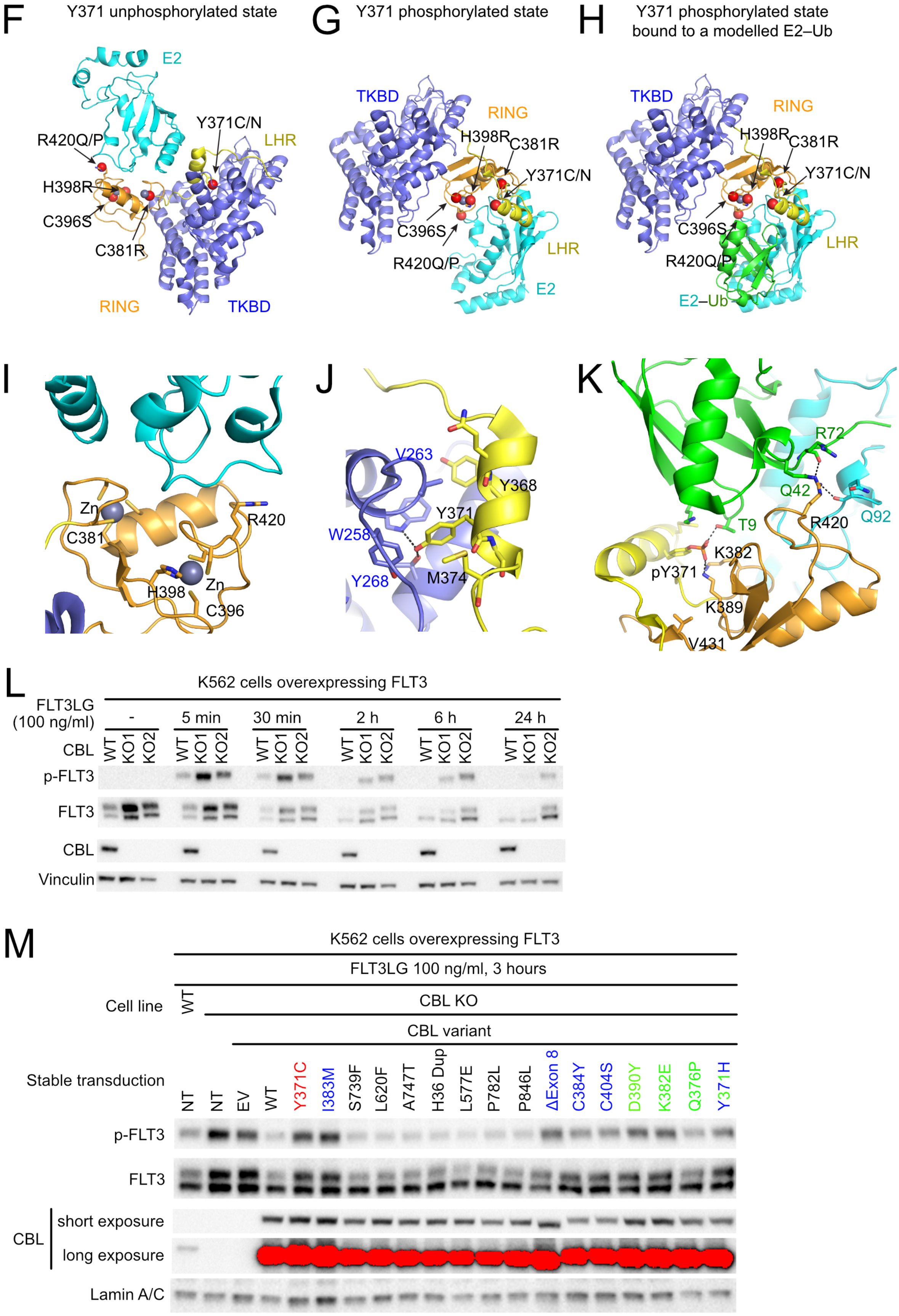

Supplemental Figure 2

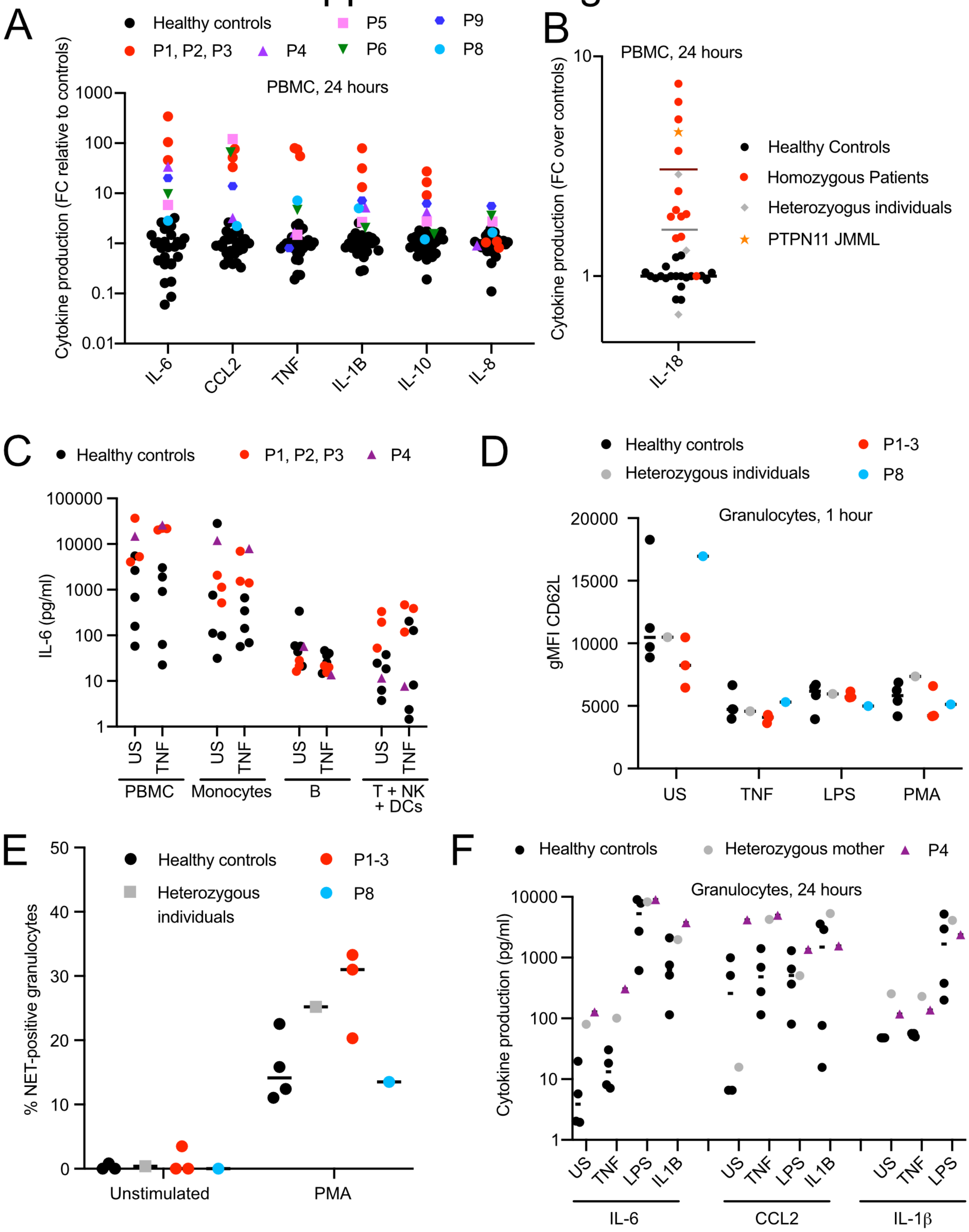

Supplemental Figure 3

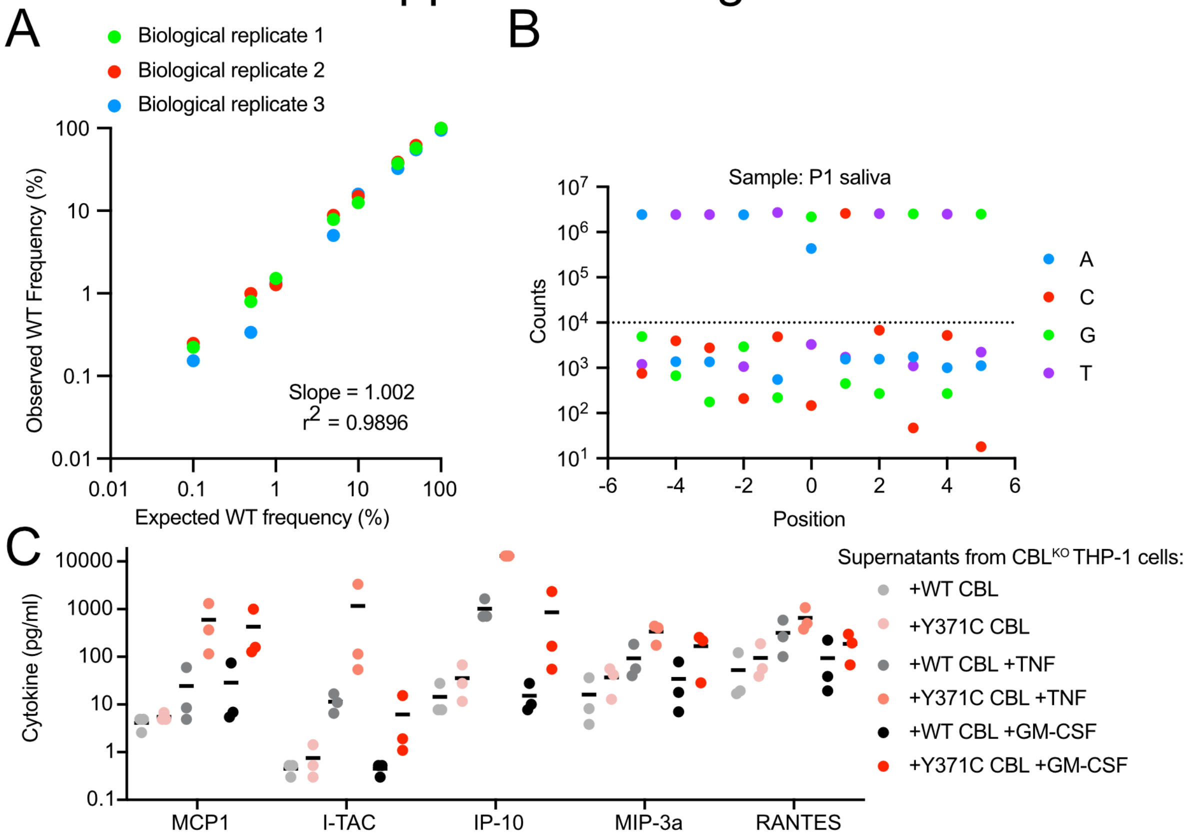

Supplemental Figure 4

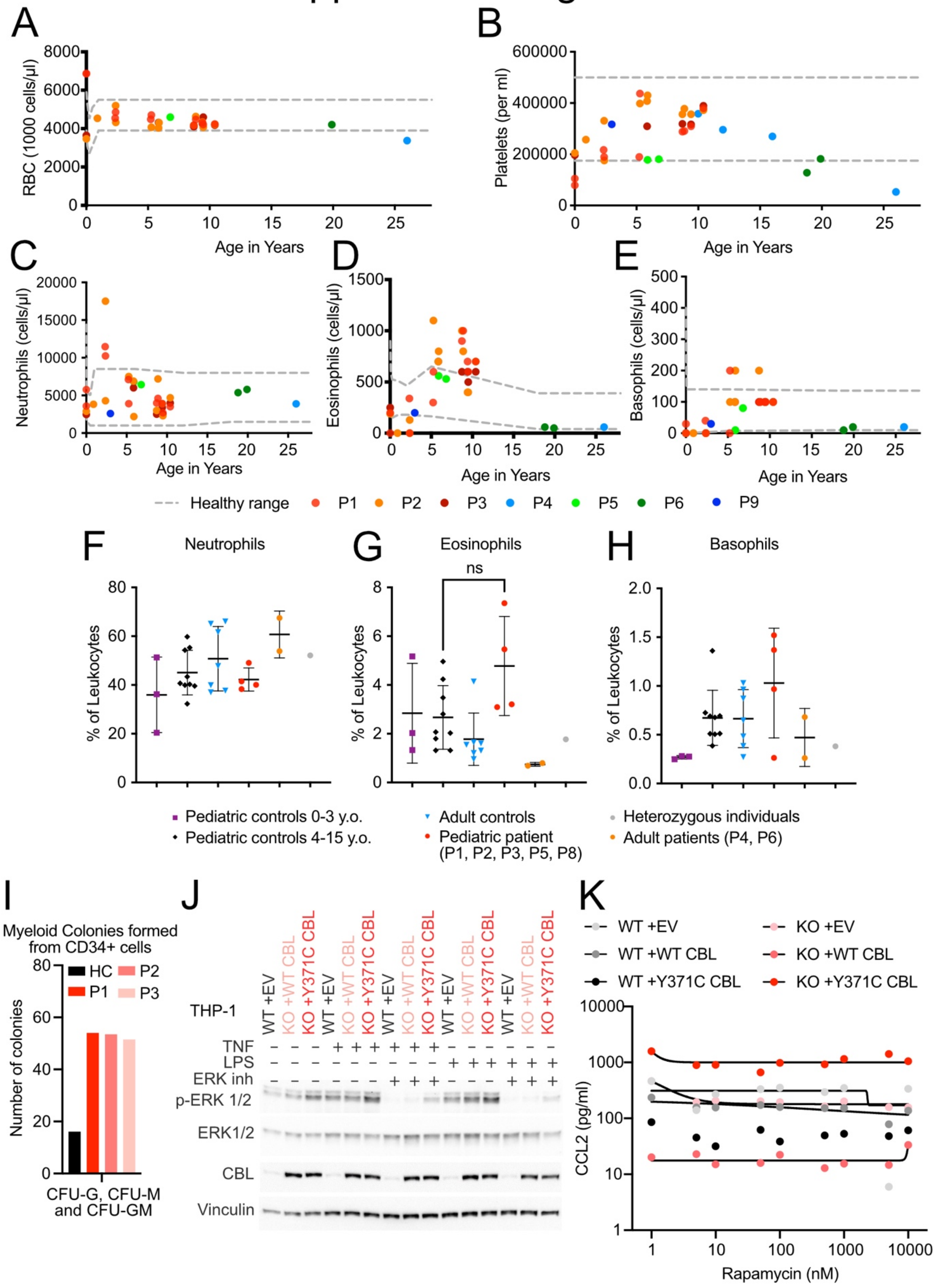

Supplemental Figure 5

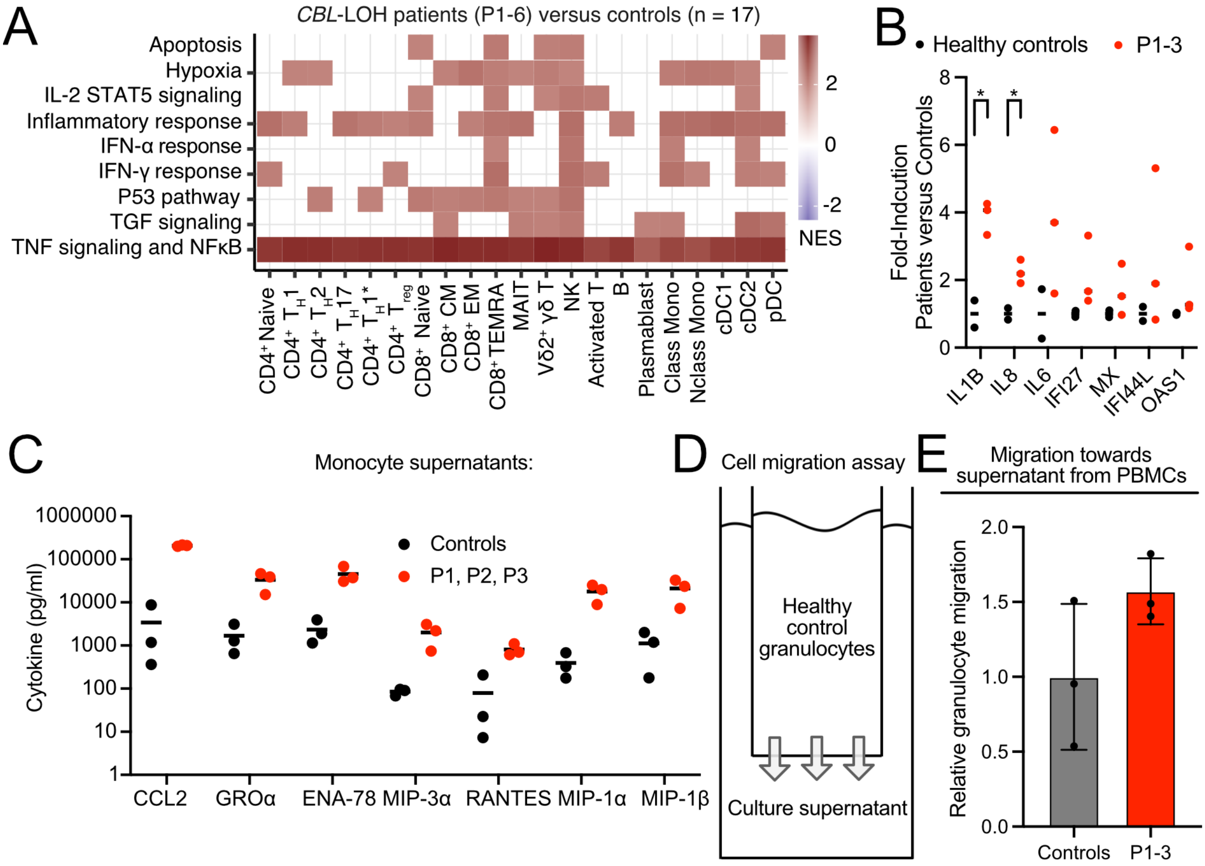

Supplemental Figure 6

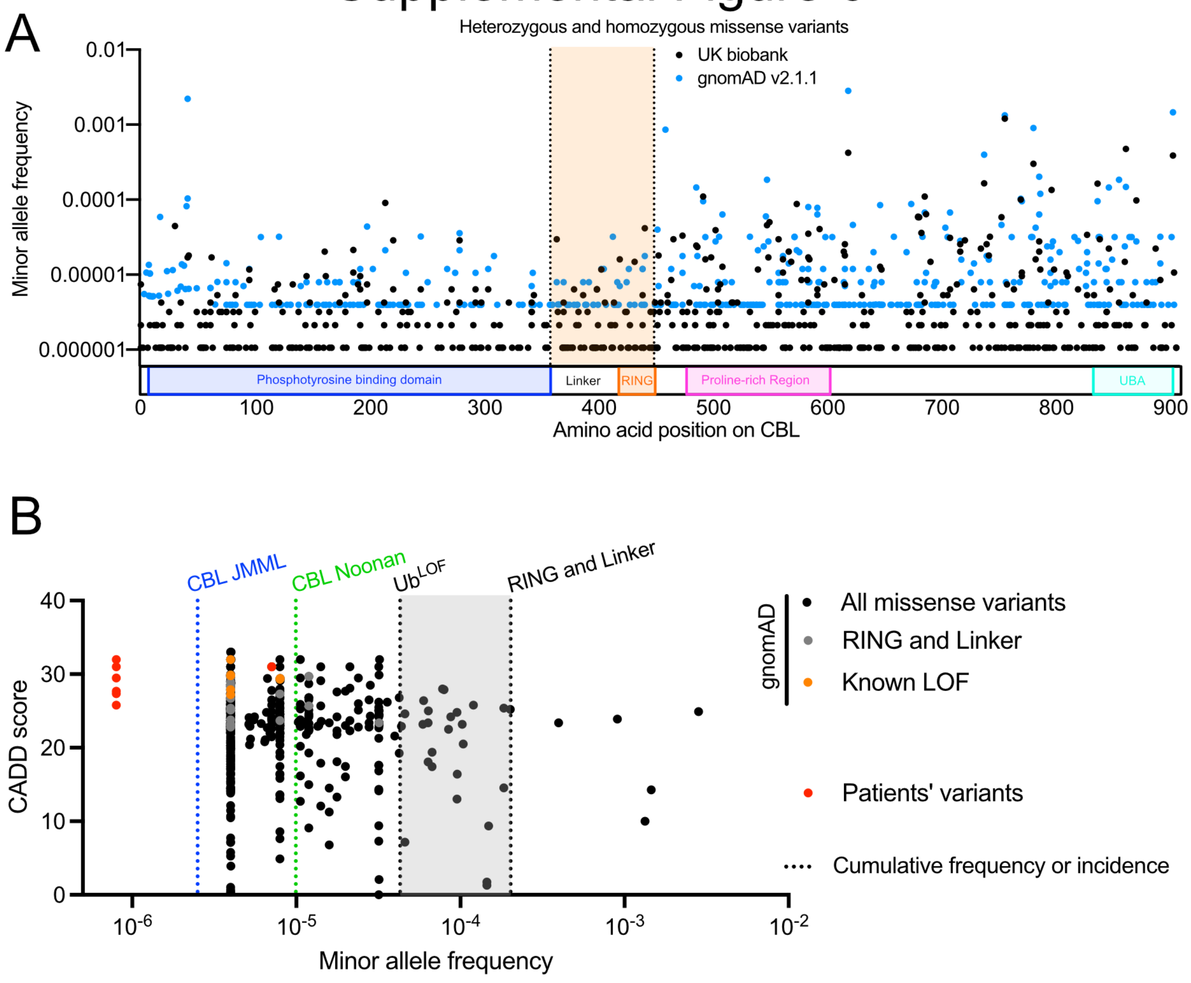

Supplement: Supplemental data [file jci-134-181604-s007.pdf]
