# Supplementary material for: Autoinflammation in patients with leukocytic CBL loss of heterozygosity is caused by constitutive ERK-mediated monocyte activation
Source: J Clin Invest. 2024 Oct 15;134(20):e181604. doi: 10.1172/JCI181604 (PMC11475086; doi:10.1172/JCI181604)
Supplement: Unedited blot and gel images [file jci-134-181604-s008.pdf]

Figure 1A

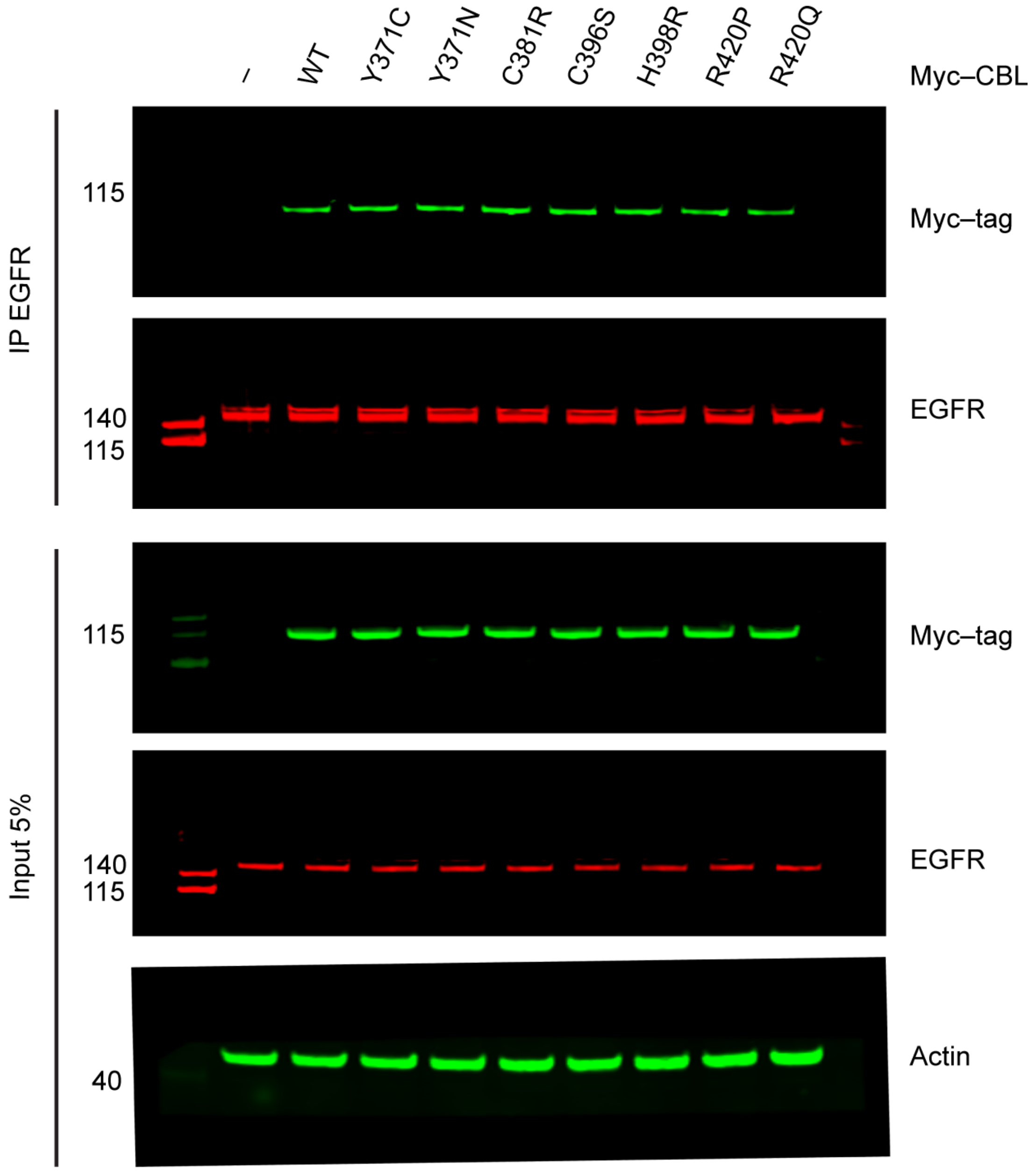

Figure 1B

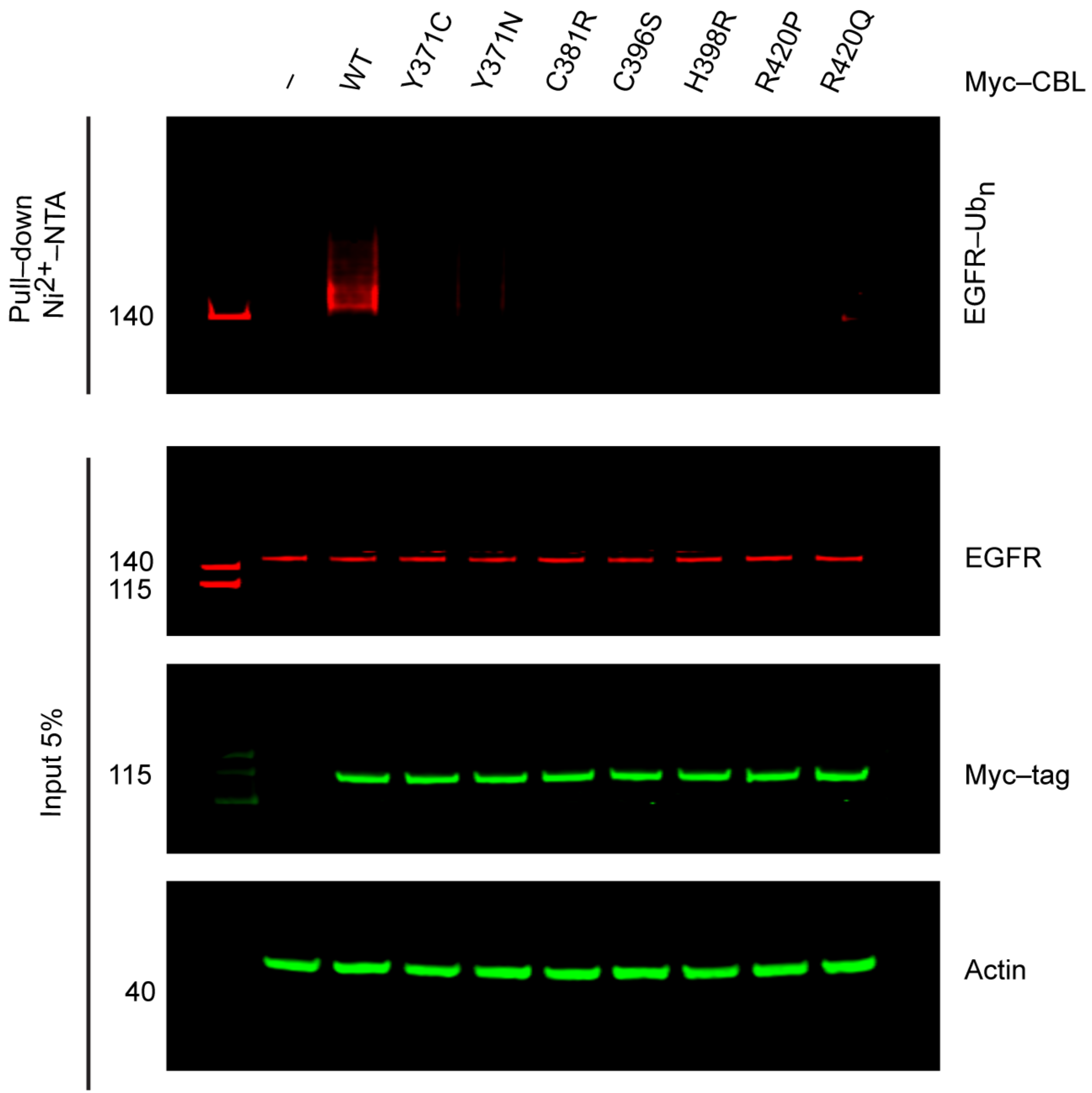

Figure 2F

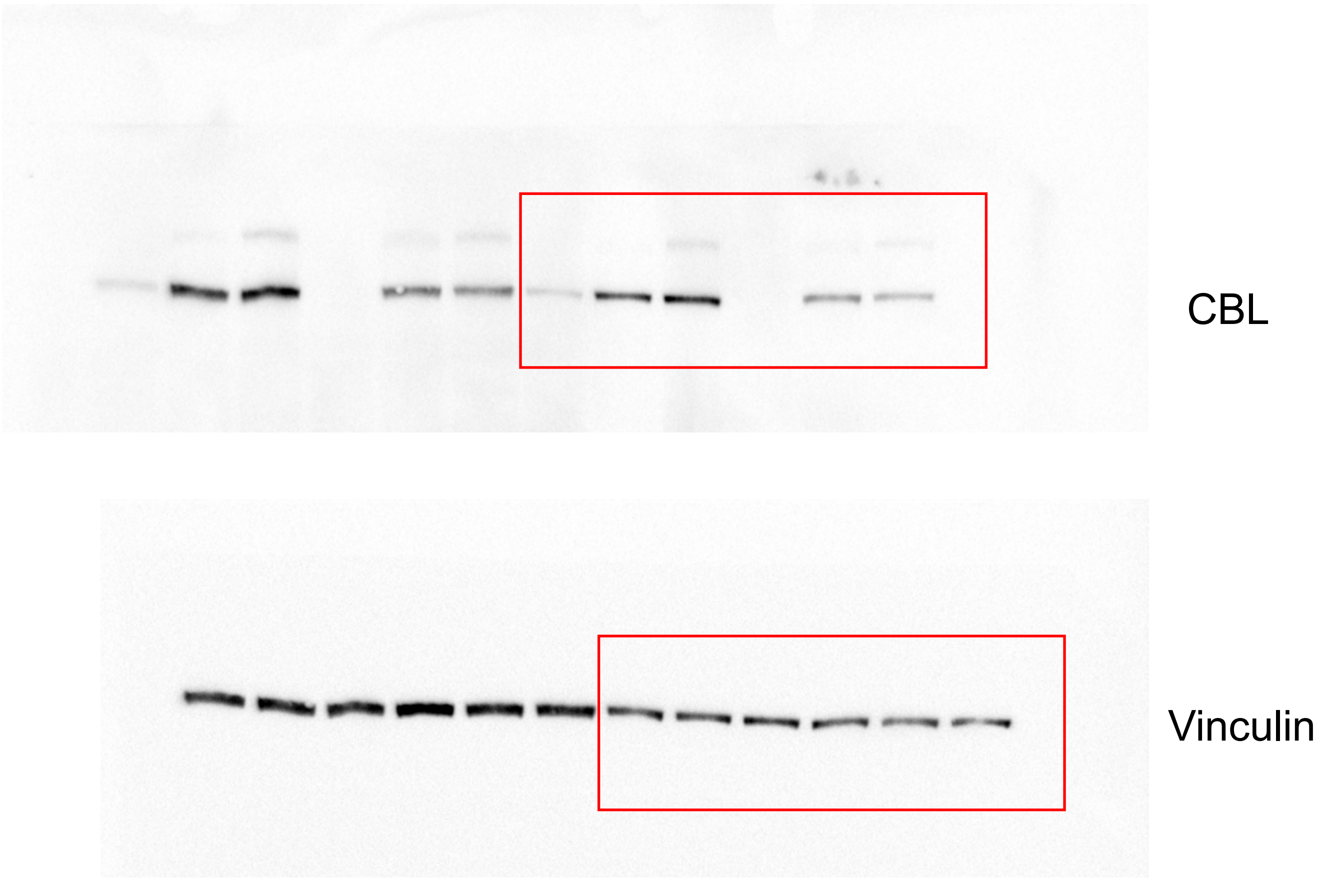

Figure S1F

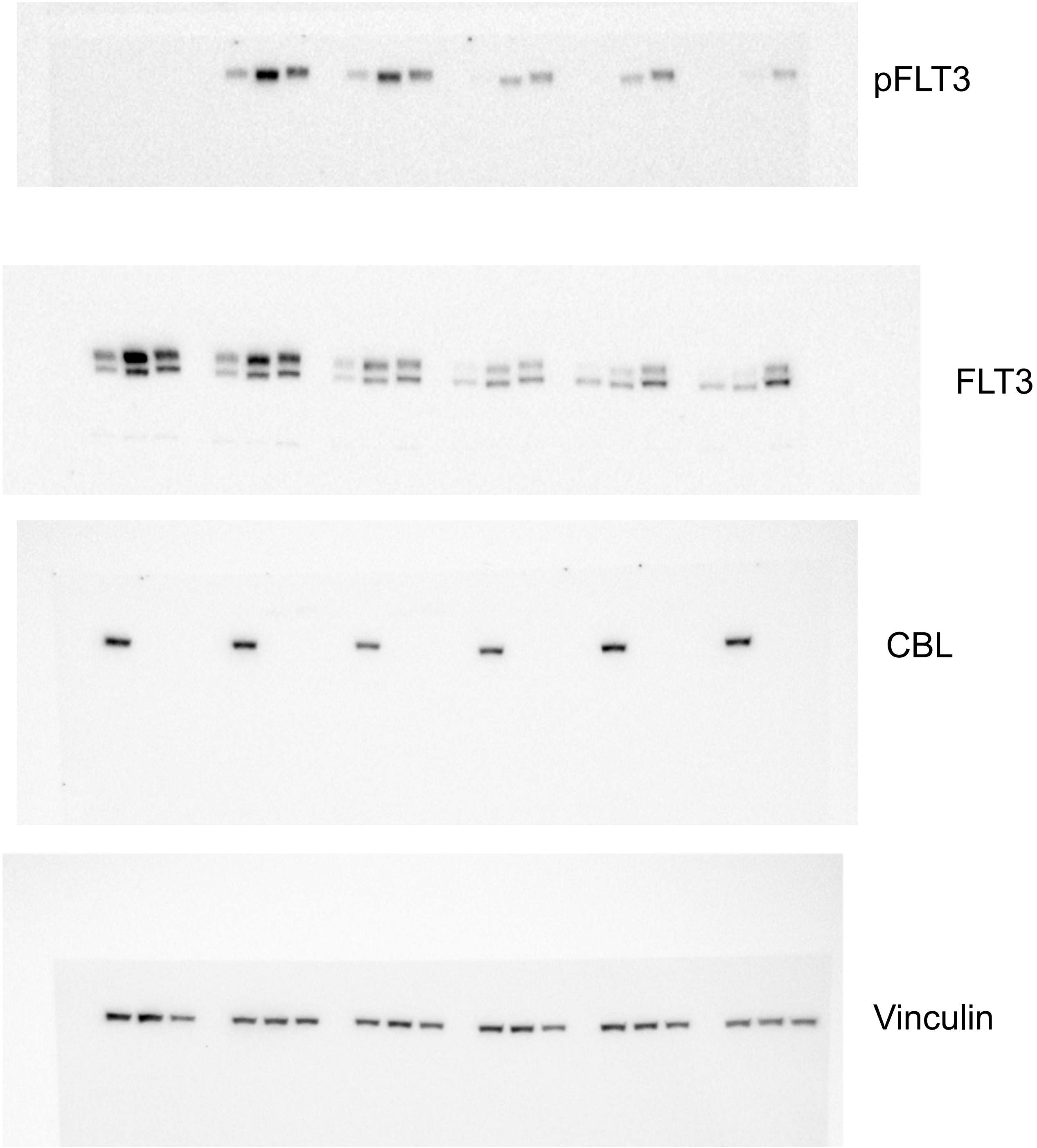

Figure S1G

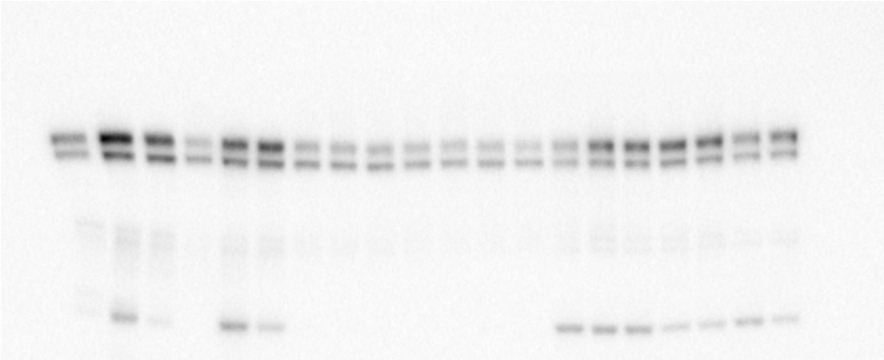

FLT3

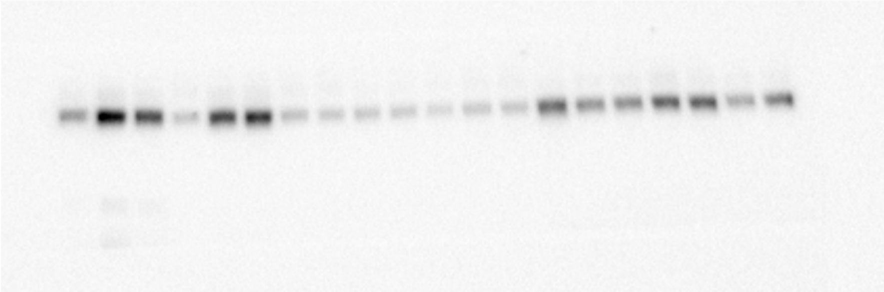

pFLT3

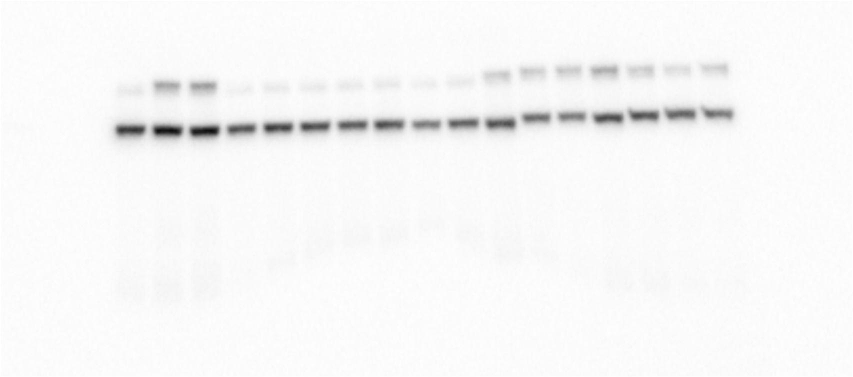

CBL

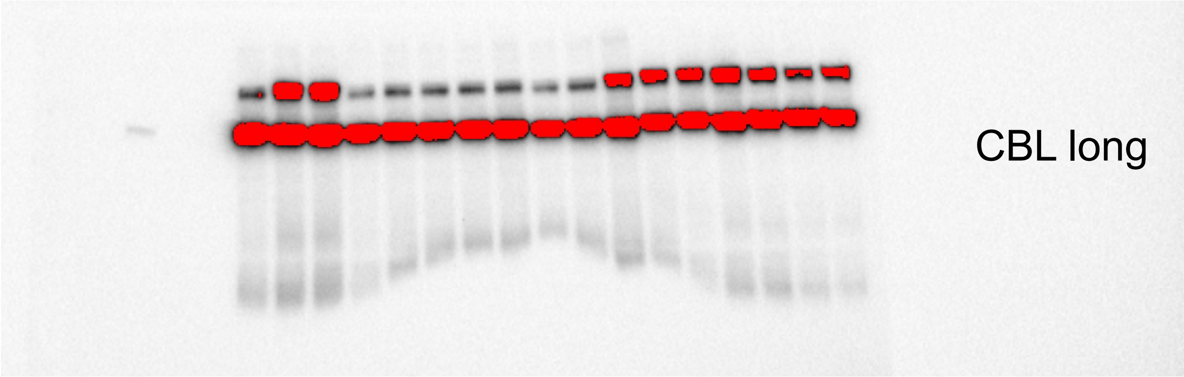

CBL long

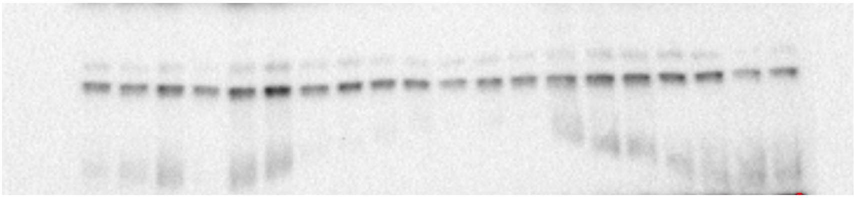

Lamin A/C

Figure S4C

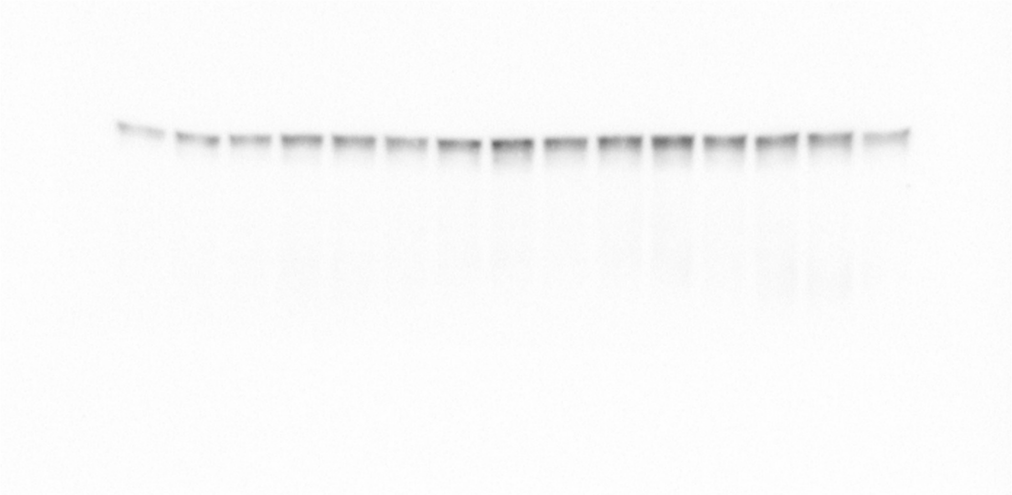
ERK

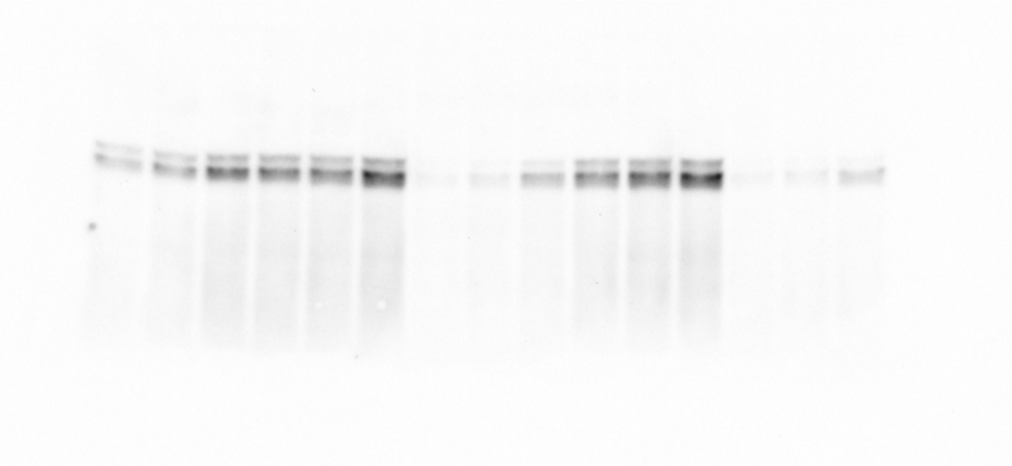
pERK

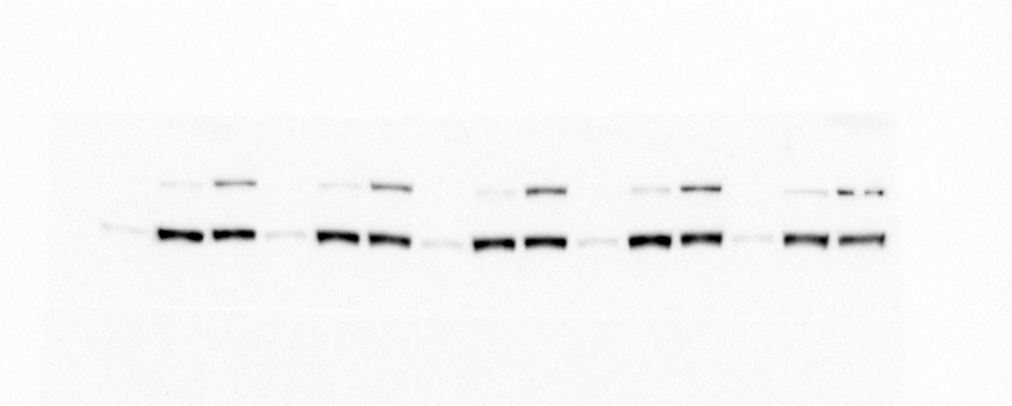
CBL

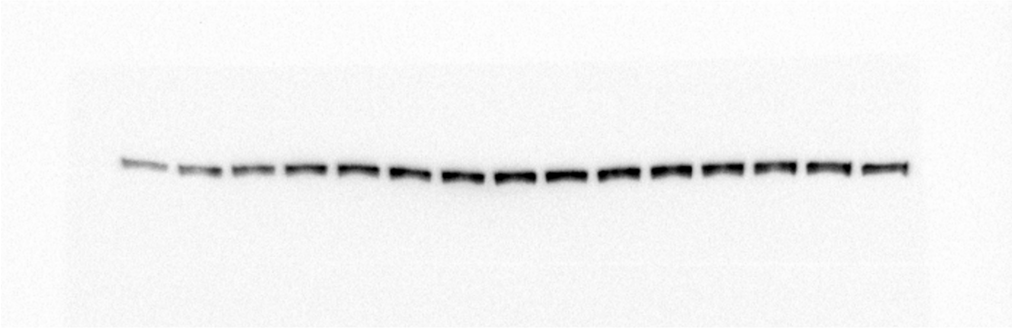
Vinculin
